# *Gemmatimonas groenlandica* sp. nov. Is an Aerobic Anoxygenic Phototroph in the Phylum Gemmatimonadetes

**DOI:** 10.3389/fmicb.2020.606612

**Published:** 2021-01-15

**Authors:** Yonghui Zeng, Nupur, Naicheng Wu, Anne Mette Madsen, Xihan Chen, Alastair T. Gardiner, Michal Koblížek

**Affiliations:** ^1^Department of Environmental Science, Aarhus University, Roskilde, Denmark; ^2^Aarhus Institute of Advanced Studies, Aarhus University, Aarhus, Denmark; ^3^Centre Algatech, Institute of Microbiology CAS, Třeboň, Czechia; ^4^Department of Geography and Spatial Information Techniques, Center for Land and Marine Spatial Utilization and Governance Research, Ningbo University, Ningbo, China; ^5^The National Research Centre for the Working Environment, Copenhagen, Denmark; ^6^Department of Engineering, Aarhus University, Aarhus, Denmark

**Keywords:** MALDI-TOF MS, bacterial isolation, phototrophy, Gemmatimonadetes, oligotrophic environment

## Abstract

The bacterial phylum Gemmatimonadetes contains members capable of performing bacteriochlorophyll-based phototrophy (chlorophototrophy). However, only one strain of chlorophototrophic Gemmatimonadetes bacteria (CGB) has been isolated to date, hampering our further understanding of their photoheterotrophic lifestyle and the evolution of phototrophy in CGB. By combining a culturomics strategy with a rapid screening technique for chlorophototrophs, we report the isolation of a new member of CGB, *Gemmatimonas (G.) groenlandica* sp. nov., from the surface water of a stream in the Zackenberg Valley in High Arctic Greenland. Distinct from the microaerophilic *G. phototrophica* strain AP64^T^, *G. groenlandica* strain TET16^T^ is a strictly aerobic anoxygenic phototroph, lacking many oxygen-independent enzymes while possessing an expanded arsenal for coping with oxidative stresses. Its pigment composition and infra-red absorption properties are also different from *G. phototrophica*, indicating that it possesses a different photosystem apparatus. The complete genome sequence of *G. groenlandica* reveals unique and conserved features in the photosynthesis gene clusters of CGB. We further analyzed metagenome-assembled genomes of CGB obtained from soil and glacier metagenomes from Northeast Greenland, revealing a wide distribution pattern of CGB beyond the stream water investigated.

## Importance

The bacterial phylum Gemmatimonadetes is an important but yet understudied group in natural microbial communities. The isolation of the only phototrophic member of this phylum, *Gemmatimonas phototrophica*, was reported in 2014, which expanded the list of known bacterial phyla capable of performing photosynthesis. Since then, no new phototrophic member of this phylum has been isolated. By applying a novel isolation strategy of combining a mass spectroscopy-based high-throughput profiling method and a rapid screening technique for phototrophic bacterial colonies, we successfully isolated the second phototrophic member of this phylum, *Gemmatimonas groenlandica*, from a stream in Northeast Greenland. Its discovery confirms the widespread presence of phototrophic Gemmatimonadetes bacteria in the environment and raises an intriguing question on the evolutionary history of phototrophy in the phylum Gemmatimonadetes. Distinct from the microaerophilic slow growth rate in *G. phototrophica*, *G. groenlandica* is a strict aerobe and can be readily cultured in liquid medium, opening new possibilities for future strain genetic engineering and detailed photophysiological studies.

## Strain Information

*Gemmatimonas groenlandica* strain TET16^T^ has been deposited to the Leibniz-Institut DSMZ-Deutsche Sammlung von Mikroorganismen und Zellkulturen GmbH, Germany under accession no. DSM110279 and to the China General Microbiological Culture Collection Center under accession no. CGMCC1.18661.

## Introduction

Members of bacterial phylum Gemmatimonadetes are widely distributed in natural microbial communities, ranked as one of the nine most abundant phyla found in soils (Janssen, 2006; Youssef and Elshahed, 2009) with a mean abundance of 2.2% of the total soil bacteria (DeBruyn et al., 2011). A more recent survey of 1,706 metagenomes from various environments that were deposited into the MG-RAST server (Wilke et al., 2016) showed that Gemmatimonadetes constitute up to 2.54% total reads with a median value of 0.24% (calculated based on the data in Supplementary Table 1 in Zeng et al., 2016). Gemmatimonadetes are most abundant in soils, wastewater treatment-related samples, biofilms, and plant-associated habitats with the largest proportion (2.54%) reported in an Arctic tundra permafrost metagenome (Zeng et al., 2016). Diversity surveys based on 16S rRNA genes indicate that Gemmatimonadetes are well adapted not only to arid but also to oligotrophic conditions (Hanada and Sekiguchi, 2014).

Despite the widespread distribution of Gemmatimonadetes in the environment, their physiology, ecology and importance in environmental processes are poorly understood (DeBruyn et al., 2011; Hanada and Sekiguchi, 2014). One critical obstacle to an improved understanding of the ecological roles of Gemmatimonadetes is that, since the establishment of the phylum Gemmatimonadetes in 2003 (Zhang et al., 2003), only a few members have been isolated as pure cultures available for detailed studies in the laboratory. To date, the validated type strains in Gemmatimonadetes (updated list accessed at^1^) include *Gemmatimonas (G.) aurantiaca* T-27^T^ isolated from activated sludge in a wastewater treatment plant (Zhang et al., 2003), *Gemmatimonas phototrophica* AP64^T^ from a desert lake (Zeng et al., 2015), *Longimicrobium terrae* CB-286315^T^ from Mediterranean forest soil (Pascual et al., 2016), and *Roseisolibacter agri* AW1220^T^ from agricultural floodplain soil (Pascual et al., 2018). Studies of this limited number of type strains have revealed some ecologically important metabolisms in Gemmatimonadetes. For instance, *Gemmatimonas aurantiaca* T-27^T^ is capable of reducing the potent greenhouse gas N_2_O under both anaerobic and aerobic conditions (Park et al., 2017; Chee-Sanford et al., 2019); *Gemmatimonas phototrophica* AP64^T^ is a microaerophilic, facultative photoheterotroph capable of harvesting light energy (Zeng et al., 2015; Koblížek et al., 2020).

Before this study, *G. phototrophica* AP64^T^ represented the only phototrophic isolate known in the phylum Gemmatimonadetes, possessing type-2 reaction centers that are possibly of proteobacterial origin based on the phylogenies of bacteriochlorophyll biosynthesis genes and the organization of its photosynthesis gene cluster (PGC) (Zeng et al., 2014). Despite the close phylogenetic relationship with purple photosynthetic Proteobacteria (Zeng et al., 2014), the PGC of *G. phototrophica* appears to display a unique feature that the *acsF* gene (involved in BChl biosynthesis pathway) is located between the *bchFNBHLM* and *puhABC* sub-clusters (see Figure 8 in Zeng and Koblížek, 2017), implying an, as yet unknown, evolutionary history of the PGC and phototrophy in CGB. More isolates of CGB are required to test whether the photosynthesis-related genomic and physiological characteristics observed in *G. phototrophica* are common features in all CGB members.

A metagenomic survey using *acsF* (encoding the Mg-protoporphyrin IX monomethyl ester oxidative cyclase) as the marker gene revealed that CGB are widely distributed in various environments, including air, river waters/sediment, estuarine waters, lake waters, biofilms, plant surfaces, intertidal sediment, soils, springs, and wastewater treatment plants, but not in marine systems (Zeng et al., 2016). The wide distribution of CGB in nature and its relatively high abundance among the phototrophic microbial community (0.4–11.9%: Zeng et al., 2016) provide enormous opportunities for the isolation of new CGB members from the environment.

In polar terrestrial environments, phototrophic bacteria have been understudied largely due to the difficulties in sampling and commonly perceived low activities of phototrophic bacteria caused by freezing temperatures and prolonged darkness in winter. A recent bacterial cultivation effort on Antarctic soils identified 330 possibly aerobic anoxygenic phototrophs (Tahon and Willems, 2017), highlighting that polar terrestrial environments could be an untapped source of novel bacterial phototrophs. In this study, we focused on the High Arctic environment in Northeast Greenland with the aim to isolate novel members of CGB. By combining a high-throughput culturomics approach (Lagier et al., 2018) with the rapid screening technique for bacteriochlorophyll-containing colonies (Zeng et al., 2014), we successfully isolated the second chlorophototrophic member of Gemmatimonadetes, *Gemmatimonas groenlandica* TET16^T^, from a stream water sample from the Zackenberg Valley. Phenotypic and genotypic comparisons of *G. groenlandica* with *G. phototrophica* allow us to reveal the unique physiological and genomic features in CGB. *G. groenlandica* strain TET16^T^ represents the first fully aerobic anoxygenic photoheterotroph in the phylum Gemmatimonadetes.

## Results and Discussion

### A Culturomics Strategy led to the Isolation of *Gemmatimonas groenlandica* sp. nov. Strain TET16^T^

**FIGURE 1:**
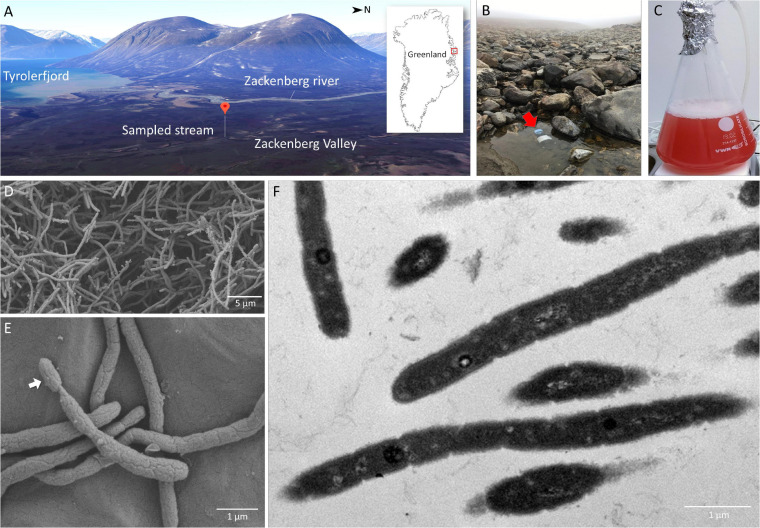
Sampling site at a stream near the Zackenberg Research Station in Northeast Greenland **(A,B)**, 2-week old pure culture of *Gemmatimonas groenlandica* strain TET16^T^ aerobically grown in a liquid medium **(C)** and microscopic images of the TET16^T^ cells (**D,E** – scanning electron microscopy; **F** – transmission electron microscopy). The 3D map view of the Zackenberg Valley facing the West was generated from Google Maps.

**FIGURE 2:**
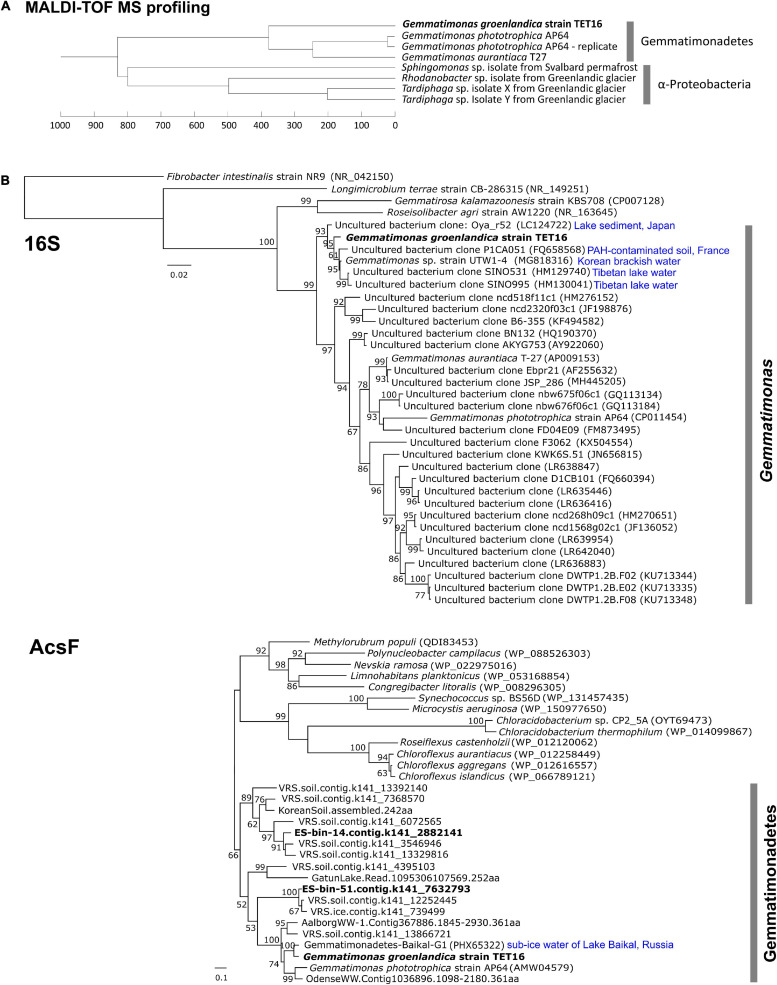
Dendrogram cluster analysis of the matrix-assisted laser desorption ionization-time of flight mass spectrometry (MALDI-TOF MS) profiles of the candidate strain TET16^T^ with reference strains from the phylum Gemmatimonadetes **(A)** and phylogenetic analyses of the 16S rRNA gene and *acsF* gene (encoding aerobic magnesium-protoporphyrin IX monomethyl ester cyclase) of the strain TET16^T^ **(B)**. MALDI-TOF MS profiles were generated from 2 weeks old colonies on a Bruker’s MALDI Biotyper system (see section “Materials and Methods”). A technical replicate of strain AP64^T^ were performed to assess variations within samples. For phylogenetic analysis, reference sequences were either downloaded from NCBI through BLAST analysis (16S and *acsF*) or retrieved from previous studies (*acsF*). The length cutoffs for reference sequences are 1,375 bp for 16S (i.e., >90% coverage) and 250 residues for *acsF* (i.e., >70% coverage). Type strains and those references closely related to strain TET16^T^ are highlighted with their source environments shown on the tree. Bars represent nucleotide (16S tree) or amino acid (*acsF* tree) substitution rates. On the *acsF* tree, tBLASTn matches from a Greenlandic glacier and soil metagenomics survey (named as VRS.ice.xxx and VRS.soil.xxx, respectively, details see section “Materials and Methods”) were included. The split *acsF* gene from LF-bin-339 was not included.

We adopted a culturomics strategy that combined a high-throughput colony screening approach using matrix-assisted laser desorption ionization-time of flight mass spectrometry (MALDI-TOF MS) and genome sequencing (Lagier et al., 2018) to search for novel Gemmatimonadetes bacteria in a stream in Northeast Greenland (Figure 1). The rapid detection technique of BChl *a* fluorescence emitted from chlorophototrophic colonies (Zeng et al., 2014) allowed us to only load BChl-containing colonies into the MALDI-TOF mass spectrometer. Based on the colony morphology of the only CGB isolate, *Gemmatimonas phototrophica* strain AP64^T^ (Zeng et al., 2015), the search focus was placed on small colonies with pink or reddish color. With a modest screening effort of ∼500 phototrophic colonies grown on heterotrophic media, we found the candidate CGB strain TET16^T^, the colonies of which appeared after 5-week incubation on 1/5 R2A agar supplemented with 8 μg/mL tetracycline. The colony appeared as a circular shape with a size of 1–2 mm. Strain TET16 was the only one in our culture collection that clustered with *G. phototrophica* and *G. aurantiaca* on the dendrogram of MALDI-TOF MS profiles, while distantly related to Alphaproteobacteria (Figure 2A).

**FIGURE 3:**
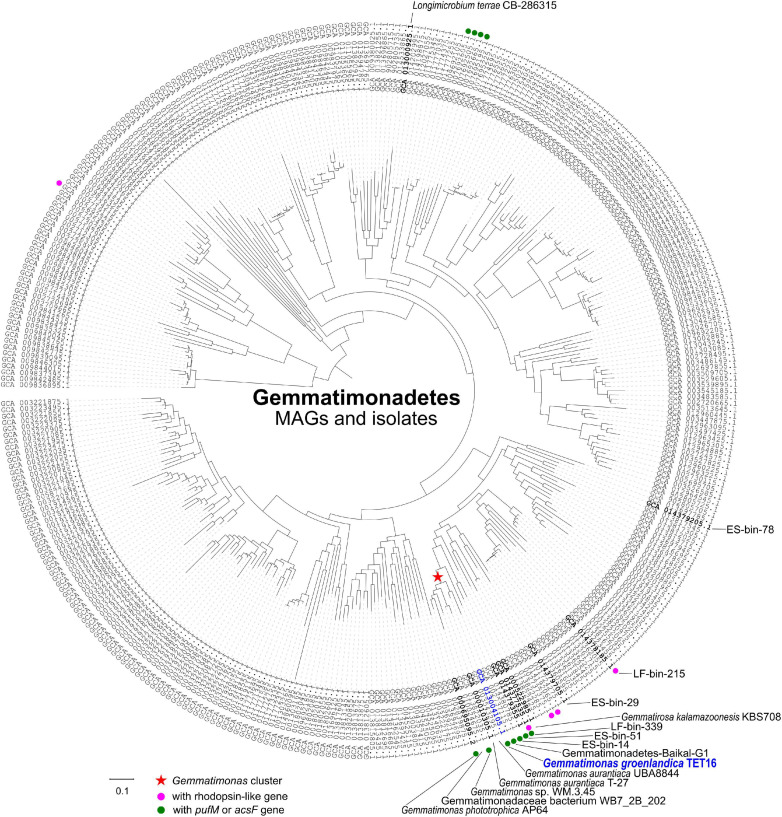
A maximum likelihood phylogenomic tree of the metagenome-assembled genomes (MAGs) and isolates of the phylum Gemmatimonadetes based on aligned 36,076 amino acid positions from 400 most universal marker genes (see section “Materials and Methods”). All 364 Gemmatimonadetes genomes were downloaded from the NCBI microbial genomes portal. The tree was rooted with the genome of *Fibrobacter succinogenes* strain S85 (GenBank assembly no. GCA 000146505.1). The *Gemmatimonas* cluster, type strains, and the MAGs discussed in this study were highlighted on the tree. Bar represents the amino acid substitution rate. The genomes with rhodopsin-like gene or *pufM*/*acsF* gene were marked to show the distribution of phototrophic Gemmatimonadetes.

The complete genome sequence of strain TET16^T^ confirmed it belongs to the phylum Gemmatimonadetes. The 16S rRNA gene phylogeny placed TET16^T^, *G. phototrophica*, and *G. aurantiaca* into the same cluster (Figure 2B). The environmental clones that clustered with *G. groenlandica* originate from various environments, including Tibetan lake water, Korean brackish water, French soil, and lake sediment in Japan (Figure 2B). A similar pattern in source environments was also observed on the environmental clones clustering with *G. phototrophica* (Zeng et al., 2014), indicating the wide distribution of CGB in natural environments. This was in line with our previous finding from a survey of public metagenomic databases that CGB were present in diverse environments (Zeng et al., 2016). The phylogenetic tree of the *acsF* gene that is involved in bacteriochlorophyll biosynthesis shows that strain TET16^T^ is more closely related to a pelagic bacterium (20 m deep) of Lake Baikal in Russia (Cabello-Yeves et al., 2018) than to *G. phototrophica* AP64^T^, which was isolated from a desert lake in North China (Figure 2B). The phylogenomic tree of all Gemmatimonadetes-affiliated metagenome-assembled genomes (MAGs) available in the NCBI Genome database and well-characterized isolates also revealed a close relationship between strain TET16^T^ and *G. aurantiaca* T-27^T^ and *G. phototrophica* AP64^T^, forming a tight cluster on the tree (Figure 3).

**FIGURE 4:**
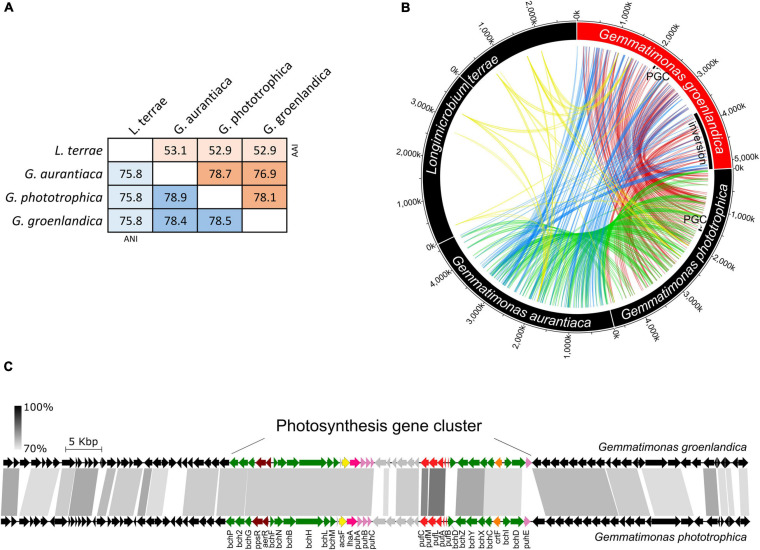
Genome-level sequence similarity between *Gemmatimonas groenlandica* TET16^T^ and the other three type strains in the phylum Gemmatimonadetes including *Gemmatimonas (G.) phototrophica* AP64^T^, *G. aurantiaca* T-27^T^, *Longimicrobium (L.) terrae* CB-286315^T^. **(A)**, average nucleotide identities (ANI) and average amino acid identities (AAI). **(B)**, genome similarity assessed by connecting highly conserved regions (>80% identity, >2 kb) between each pair. Note that the *L. terrae* genome contains two chromosomes. Any plasmid that may exist was excluded from the analysis. The loci of photosynthesis gene clusters (PGCs) in *G. groenlandica* and *G. phototrophica* are marked. An inversion event of a large genomic region in *G. groenlandica* was identified and marked. **(C)**, Conserved genome synteny of the PGCs (∼42 kb) and neighboring regions (∼30 kb in each side; see details in Supplementary Figure 1) in *G. groenlandica* and *G. phototrophica*. Connecting gray blocks represent matched regions by BLASTn analysis with the cutoffs of >70% sequence identity and >500 bp in length.

Strain TET16^T^ and the two type strains of the same genus, *G. phototrophica* AP64^T^ and *G. aurantiaca* T-27^T^, share 78.4∼78.9% average nucleotide identity (ANI) and 76.9∼78.7% average amino acid identity (AAI) (see Figure 4A). The ANI is lower than the threshold of 95∼96% proposed for species delimitation (Kim et al., 2014; Rosselló-Móra and Amann, 2015), while the 16S rRNA gene of strain TET16^T^ shares 95.7% identity to *G. phototrophica* AP64^T^ and 95.9% identity to *G. aurantiaca* T-27^T^, above the 95% threshold for defining a new genus but below the 98.7% threshold for defining a new species (Chun et al., 2018). Furthermore, the phylogenomic analysis of all existing Gemmatimonadetes isolates and MAGs in the NCBI genome database showed that strains TET16^T^, AP64^T^, and T-27^T^ were closely related to each other, forming the tight *Gemmatimonas* cluster (Figure 3). The genome-based taxonomic tool GTDB-Tk also classified TET16^T^ into the *Gemmatimonas* genus with a relative evolutionary divergence (RED) value of 0.934 at the genus level, which was higher than the median RED value of 0.902 calculated from all genera in the database. These lines of evidence, together with the high genome-level synteny between strain TET16^T^ and the other two *Gemmatimonas* species (Figure 4B), supports that strain TET16^T^ represents a new species within the genus *Gemmatimonas*, named *Gemmatimonas groenlandica*, with its source location of Greenland (Groenland in Danish) designated as the species name.

Slow growth and formation of tiny colonies are common characteristics of the hitherto cultured Gemmatimonadetes bacteria (see summarized characteristics in Pascual et al., 2018). Thus, they can be easily outcompeted by fast growers during initial enrichment from the environment, which largely explains why there are so few pure cultures formally described in the phylum Gemmatimonadetes. Some technological innovations have been introduced to circumvent these issues. For example, the use of a diffusion sandwich system consisting of an array of 384 miniature diffusion chambers and a sample dilution-based high-throughput approach successfully led to the isolation of *Longimicrobium terrae* (Pascual et al., 2016) and *Roseisolibacter agri* (Pascual et al., 2018), respectively. In this study, we show the use of antibiotics is also an effective means to recover Gemmatimonadetes diversity in pure cultures. Although *G. groenlandica* strain TET16^T^ was initially isolated from an agar plate supplemented with tetracycline, our antibiotics susceptibility test showed that, interestingly, its growth was completely inhibited by tetracycline. Its genome also lacks genes for degrading tetracycline or transporting tetracycline outside the cell. It is likely that during the initial incubation, tetracycline inhibited the growth of bacterial cells surrounding TET16^T^ cells, and the ensuring depletion of tetracycline by these surrounding cells created a favorable micro-niche for TET16^T^ to grow in the area where tetracycline was removed through passive diffusion. Given the high light sensitivity of tetracycline, the other possibility is that tetracycline initially inhibited fast growers but over time was photo-degraded, and then the slow growing TET16^T^ cells started to propagate.

### *Gemmatimonas groenlandica* Is an Aerobic Anoxygenic Phototroph

In contrast to the microaerophilic *G. phototrophica* AP64^T^, *G. groenlandica* TET16^T^ grows well in liquid T21 medium under fully aerobic conditions (reaching stationary phase within 7∼10 days). It can also grow under microaerophilic conditions (∼10% O_2_) albeit much more slowly. Fermentative growth was not observed under anaerobic conditions. Growth did not occur under photoautotrophic and chemoautotrophic conditions using sulfide and thiosulfate as electron donors and NaHCO_3_ as carbon source. Therefore, chemoorganoheterotrophic and photoheterotrophic are the preferred growth modes with the ability to utilize various carbon sources (Table 1) under aerobic, light or dark conditions.

**TABLE 1:** Comparison of phenotypic, physiological and genomic characteristics of the two phototrophic members in the phylum Gemmatimonadetes, *Gemmatimonas groenlandica* strain TET16^T^ and *Gemmatimonas phototrophica* strain AP64^T^.

	*G. groenlandica* TET16^T^	*G. phototrophica* AP64^T^
**Cell**		
shape	short to long rod	short to long rod
color	pink to red	red
capsule-like structure	yes	yes
fission mode	binary, budding	binary, budding
**Genome**		
GC%	65.1%	64.4%
size (Mbp)	5.179	4.717
plasmid	n. d.	n. d.
**Growth**		
temp. range	15–32°C	20–30°C
pH range	6.5–9.0	6.0–9.0
pH optimum	7.3	7.5–8.0
oxygen requirement	fully aerobic, 10% O_2_ tolerant	obligate microaerophilic
carbon source	yeast extract, saccharin, salicin, adonitol, trehalose, dulcitol, rhamnose, pyruvate, glucose	yeast extract
non-utilized carbon source	xylose, ribose, erythritol, turanose, cellobiose, melibiose, lyxose, arabinose	casamino acids, sodium succinate, sodium acetate, sodium pyruvate, potato starch, sucrose, L-glutamic acid, L-leucine, L-arginine, L-alanine, L-isoleucine, L-arabinose, D-sorbitol, D-mannitol
resistant to antibiotics	bacitracin, chloramphenicol, nystatin	ampicillin, penicillin, paramycin sulfate, polymyxin B sulfate, nystatin
susceptible to antibiotics	neomycin, amoxicillin, tetracycline, amphotericin B	neomycin, vancomycin, bacitracin, gentamicin
in liquid media	yes	not observed
in prolonged darkness	yes*	yes
Chemotaxonomy**		
fatty acid	C15:0 iso, C15:1 ω6c	C16:1, C14:1, C18:1 ω9c
polar lipid	Phosphatidylethanolamine, aminolipid, diphosphatidylglycerol	–
quinone	MK-8, MK-9	MK-8

Data from strain AP64^T^ were retrieved from previous studies (Zeng et al., 2014, 2015). n. d., not detected. *observed for a period of 3 weeks. **only major compositions are reported.

**FIGURE 5:**
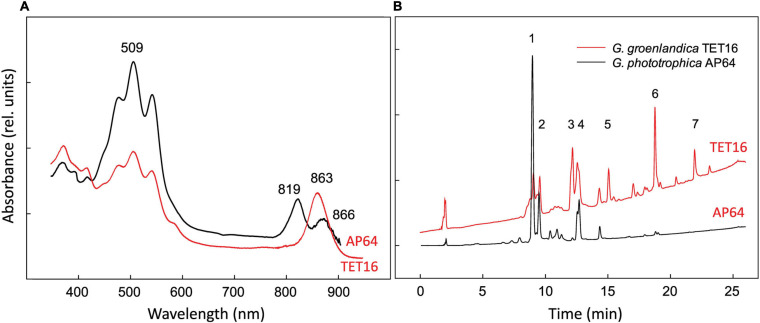
Comparison of *in vivo* absorption spectra **(A)** and HPLC elution profiles of pigments recorded at 490 nm **(B)** of *Gemmatimonas groenlandica* TET16^T^ and *Gemmatimonas phototrophica* AP64^T^. Identified peaks: 1 and 2, putative (2S,2′S)-oscillol 2,2′-di-α-L-rhamnoside; 3: unknown carotenoid, λ_max_ = 317,(470),495,527 nm; 4: unknown keto-carotenoid, λ_max_ = 499 nm; 5–6: spirilloxanthin-like carotenoids, λ_max_ = 315,(470),493,526 nm; 7: unknown carotenoids, λ_max_ = 470,500 nm.

At the genome level, phototrophic *G. groenlandica* and *G. phototrophica* appear to be more distantly related than *G. phototrophica* and non-phototrophic *G. aurantiaca* as a large inversion only occurred in *G. groenlandica* (Figure 4B), which may suggest a distinct evolutionary history in the local CGB in Greenland. Nonetheless, *G. groenlandica* and *G. phototrophica* show identical organization of photosynthesis-related genes in their PGCs with high DNA sequence identities (70–100%) (Figure 4C), implying that they share a common ancestor and, therefore, probably similar structures and properties in their reaction centers. However, surprisingly, the two species show different *in vivo* absorption spectra in the near infra-red range. Two peaks (819 and 866 nm) occurred in *G. phototrophica* corresponding to its double concentric ring of LH complexes (Dachev et al., 2017). However, only one absorption peak (863 nm) appears in *G. groenlandica* (Figure 5A). This indicates that the light harvesting system in *G. groenlandica* may have a different structure, as further evidenced by the difference in their major carotenoid composition, where the putative pigment (2*S*,2′*S*)-oscillol 2,2′-di-(α-L-rhamnoside) that dominates in *G. phototrophica* only constitutes a minor fraction in *G. groenlandica* (Figure 5B).

**FIGURE 6:**
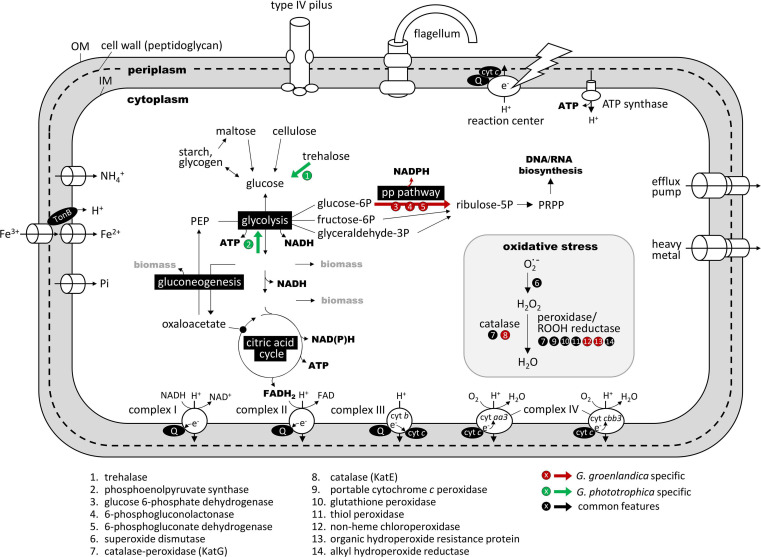
Comparison of key metabolic pathways and functions related to the aerobic photoheterotrophic lifestyle in *Gemmatimonas groenlandica* and *Gemmatimonas phototrophica* based on genome annotations. The comparison is based on the “KEGG metabolic pathway reconstruction” function of the RAST (Rapid Annotations using Subsystems Technology) web server (https://rast.nmpdr.org/; Aziz et al., 2008). A complete set of shared and unique genes between these two genomes is shown in Supplementary Table 1. OM, outer membrane; IM, cytoplasmic membrane; PRPP, phosphoribosyl diphosphate; Q, quinone pool; *cyt*, terminal cytochrome oxidase; PEP, phosphoenolpyruvate; PRPP, phosphoribosylpyrophosphate; PP pathway, pentose phosphate pathway. Red arrow indicates the pathway that only occurs in *Gemmatimonas groenlandica* and green arrows are those only present in *Gemmatimonas phototrophica*. Pathways and functions drawn in black and white are predicted in both genomes. Note that the respiratory complex IV has two types of cytochrome *c* as terminal electron acceptor. The *aa3*-type has a low affinity for O_2_ and the *cbb3*-type is a high-affinity version.

In line with the preferred aerobic lifestyle of *G. groenlandica*, its genome only contains the aerobic version of Mg-protoporphyrin IX monomethyl ester oxidative cyclase (*acsF*) and lacks the anaerobic version encoded by the *bchE* gene, whereas both *acsF* and *bchE* genes exist in the genome of *G. phototrophica* (Table 2). Similarly, the anaerobic version of coproporphyrinogen oxidase (*hemN*) that is involved in BChl biosynthesis pathway is also absent in *G. groenlandica*. Additionally, none of the denitrification genes that are commonly involved in bacterial anaerobic respiration, including nitrate reductase, nitrite reductase, nitric oxide reductase, and nitrous oxide reductase, were found in its genome. To cope with oxidative stresses associated with the aerobic lifestyle, *G. groenlandica* possesses an expanded gene repository for scavenging reactive oxygen species compared to *G. phototrophica* (Table 2 and Figure 6), including catalase (KatE), superoxide dismutase [Cu-Zn SOD], chloroperoxidase, and organic hydroperoxide resistance gene, as well as bacteriophytochrome for fine regulation of cellular metabolisms in response to light, which is one of the major causes for the generation of radical oxygen species inside cells. The limited arsenal for handling oxidative stresses in *G. phototrophica* could explain its sluggish or halted growth when exposed to fully aerobic conditions (Zeng et al., 2015).

**TABLE 2:** Key differences in the presence of genes related to bacteriochlorophyll (BChl) biosynthesis (only including three enzymes that have both aerobic and anaerobic versions) and genes related to oxidative stress response between *Gemmatimonas phototrophica* and *Gemmatimonas groenlandica*.

Functional category		Gene	Presence and GenBank access.	
Enzyme	Function		no.	
			*G. phototrophica*	*G. groenlandica*
**BChl biosynthesis pathway**				
Coproporphyrinogen oxidase	Coproporphyrinogen III → Protoporphyrinogen IX	*hemF* (aerobic) *hemN* (anaerobic)	WP_075071451 WP_075071577	QJR35625 *n. d.*
Protoporphyrinogen oxidase	Protoporphyrinogen IX → Protoporphyrin IX	hemJ* (aerobic) *hemG* (anaerobic)	WP_026851012 WP_053334334	QJR36952 QJR35626
Mg-protoporphyrin IX monomethyl ester oxidative cyclase	Mg-protoporphyrin IX → Divinylprotochlorophyllide	*acsF* (aerobic) *bchE* (anaerobic)	WP_082821546 WP_026851009	QJR35611 *n. d.*
**Oxidative stress response**				
Bacteriophytochrome, two-component sensor histidine kinase	Light sensing, regulation	*BphP*	*n. d.*	QJR35953 QJR37607
Bacteriophytochrome, heme oxygenase	Biosynthesis of the biliverdin chromophore	*BphO*	*n. d.*	QJR37606
Superoxide dismutase [Cu-Zn]	reactive oxygen species scavenging	CuZnSOD	*n. d.*	QJR38173
Superoxide dismutase [Mn]	reactive oxygen species scavenging	MnSOD	WP_169804576	QJR34760

A complete set of shared and unique genes is presented in Supplementary Table 1. *n.d.*, gene homolog not detected. *previously annotated as *hemY*, revised as *hemJ* according to Kato et al. (2010).

Metabolic reconstruction based on genome sequences reveals that *G. groenlandica* is an aerobic anoxygenic phototroph. In addition to the complete PGC and various oxidative stress response genes, it has complete sets of genes for glycolysis and citric acid cycle for generating ATP and NAD(P)H as well as respiratory complexes I-IV for generating a proton gradient across the cytoplasmic membrane (Figure 6). It also possesses genes encoding type IV pili and flagella. The major difference between *G. groenlandica* and *G. phototrophica* was the presence of the pentose phosphate pathway in *G. groenlandica*, which could potentially provide it with additional NADPH. *G. phototrophica* appears to be better equipped for carbon storage than *G. groenlandica* by possessing phosphoenolpyruvate synthase for gluconeogenesis. Despite that growth was not observed under low oxygen tension (<1%) conditions in both species, their respiratory complex IVs have both high-affinity (*aa3*-type) and low-affinity (*cbb3*-type) terminal cytochrome *c* oxidase (*cyt c*) (Figure 6). The high-affinity *cyt c* is likely used to meet basic cellular needs for energy when facing microaerobic conditions.

Aerobic anoxygenic phototrophs (AAPs) is a functional group of bacteria widely distributed in natural environments, utilizing cyclic photophosphorylation to generate ATP without the need for external electron donors and obtaining organic carbon sources from the environment (Koblížek, 2015). Distinct from their purple photosynthetic relatives, AAPs are unique in their capacity to synthesize BChl *a* exclusively under aerobic conditions. All members of AAPs identified to date belong to the phylum Proteobacteria. The discovery of the fully aerobic phototroph of *G. groenlandica* expands AAPs further into the phylum Gemmatimonadetes. The structure and physical properties of the reaction centers in *G. groenlandica* and its photophysiology warrant further investigations, for instance, how light contributes to the growth of TET16^T^ and how light influences its carbon metabolisms.

### Unique but Conserved Photosynthesis Gene Cluster in CGB

**FIGURE 7:**
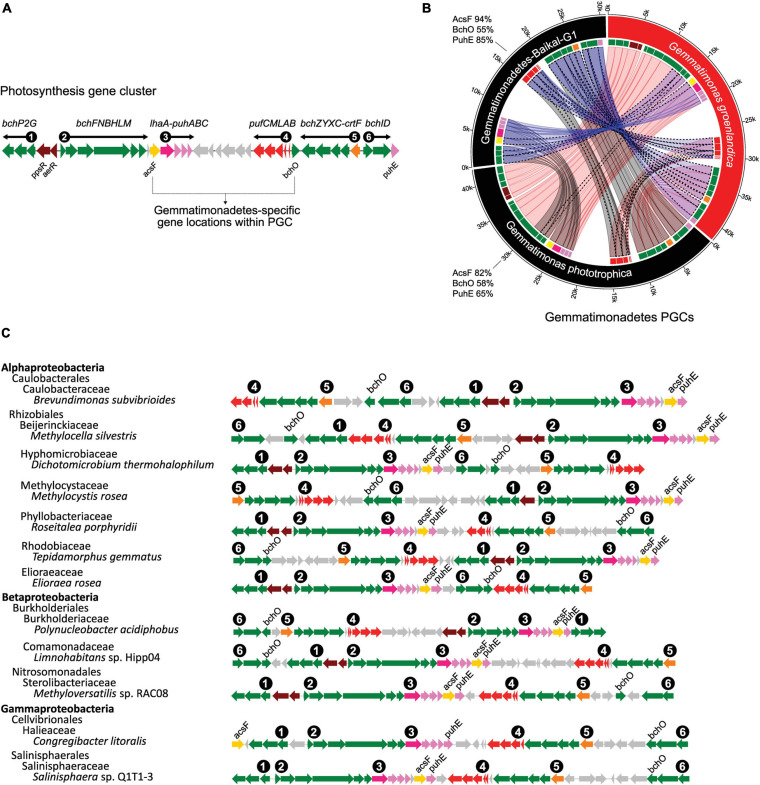
Conserved and unique gene organization of photosynthesis gene cluster (PGC) in chlorophototrophic Gemmatimonadetes bacteria. **(A)**, six gene sub-clusters commonly found in photosynthesis gene clusters. *bch* and *acsF*, bacteriochlorophyll biosynthesis genes; *puf*, genes encoding reaction center proteins; *puh*, genes encoding reaction center assembly proteins; *crt*, carotenoid biosynthesis genes; *lhaA*, light-harvesting complex I assembly protein; *ppsR* and *aerR*, regulation related genes. Gray-colored genes are hypothetical ORFs with unknown function. The unique locations of *acsF* and *bchO* are highlighted. **(B)**, comparison of three known Gemmatimonadetes PGCs including two isolates (*G. phototrophica* and *G. groenlandica*) and a MAG, Gemmatimonadetes-Baikal-G1, reconstructed from a metagenome of the Baikal Lake in Russia (Cabello-Yeves et al., 2018). The same genes are connected by ribbons. A dotted outline of a ribbon represents >90% protein sequence identity for the gene pair. The protein sequence identities of AcsF, BchO and PuhE in reference to *G. groenlandica* are shown next to the species arc. The fragmented Gemma-PGCs in the MAGs of this study were not included. **(C)** Representative PGC architecture in various proteobacterial genomes supporting the uniqueness of PGC in Gemmatimonadetes. Genomes were top tBlastn hits against NCBI’s RefSeq genome database using fused AcsF-BchO-PuhE protein sequences (see section “Materials and Methods”). One genome from each group at the class level was downloaded from NCBI. Only the top scoring genome from each class was kept as a representative.

The discovery of the second CGB member *G. groenlandica* enabled us to identify the common and unique features in the photosynthesis gene clusters of CGB (Figure 7A). The PGCs of *G. groenlandica* and *G. phototrophica* are identical in terms of gene content and organization and sub-cluster orientation, including the hypothetical genes located between the *puh* (reaction center assembly proteins) and *puf* (reaction center proteins) operons (Figure 4C). Similar patterns in operon organization were observed in some incomplete Gemmatimonadetes PGCs reconstructed from active sludge metagenomes (Zeng et al., 2016) and a Lake Baikal’s surface water metagenome (Cabello-Yeves et al., 2018). The PGC of *G. groenlandica* appear to be more closely related to MAG Gemmatimonadetes-Baikal-G1 (Cabello-Yeves et al., 2018) since their PGCs share a higher number of genes that are of >90% amino acid identity (Figure 7B), including *acsF*, *bchCDHILXYZ*, *crtF*, and *pufLMC*. In contrast, only *bchL* and *pufLMA* genes between *G. groenlandica* and *G. phototrophica* and *pufL* gene between *G. phototrophica* and the Baikal MAG are >90% identical. Together, this evidence indicates that CGB share a conserved PGC likely originating from a common ancestor.

Gemmatimonadetes PGCs (Gemma-PGCs) contain the same gene sub-clusters as Proteobacteria, including *bchP2G*, *bchFNBHLM*, *lhaA*-*puhABC*, *pufBALMC*, *crtF-bchCXYZ*, and *bchID*, but they differs in their orientations (Figure 7C). No proteobacterial PGC was found to show completely identical orientations of each sub-cluster and the same relative positions of these six sub-clusters to those in Gemma-PGC. The relative location of *acsF* and *bchO* genes are also unique in Gemma-PGC with *acsF* consistently located between the *bchFNBHLM* and *lhaA-puhABC* sub-clusters and *bchO* between *pufBALMC* and *bchCXYZ*. As environmental metagenomics data are exploding, these unique features could serve as convenient markers for identifying Gemma-PGC from metagenomic contigs.

### Aerobic CGB Also Exist in Northeast Greenland’s Soil and Glacier

Quantification of aerobic CGB in environmental samples remains a challenge. Given the high synteny and high sequence identities of the PGCs of *G. groenlandica* and *G. phototrophica*, it is practically impossible to distinguish between aerobic CGB represented by *G. groenlandica* and microaerophilic CGB like *G. phototrophica* using conventional approaches that rely on biomarker gene phylogenies. Instead, based on our comparison of anaerobic BChl biosynthetic genes and oxidative stress response genes in these two species (see above), we propose that the lack of *bchE* and presence of *BphP* and *BphO* could be strong indicators of a query genome belonging to aerobic CGB.

We applied these criteria and examined the six Gemmatimonadetes MAGs previously assembled from 460G-base shotgun reads of a surface soil sample (with prefix ES-bin) and a glacial ice sample (with prefix LF-bin) near the Villum Research Station (81°36′ N, 16°40′ W) in Northeast Greenland (Zeng et al., 2020). Three bins (ES-bin-14, ES-bin-51, and LF-bin-339) were found to contain at least one *puf* gene, one *puh* gene, and one *bch* gene (Table 3), indicating they belong to CGB. All the three MAGs possess *acsF*, which is, however, split into two fragments in LF-bin-339, whereas all lack the *bchE* gene. ES-bin-51 and LF-bin-339 contain both *BphP* and *BphO*. Despite the small dataset and the incompleteness of the MAGs (Table 3), these lines of evidence may suggest that aerobic CGB are more prevalent than microaerophilic CGB in the environment, consistent with the hypothesis that aerobic CGB can generate energy more efficiently and thus may cope more effectively with cold and nutrient stresses in supraglacial environments.

**TABLE 3:** Metagenome-assembled genomes (MAGs) of Gemmatimonadetes origin from the “Lille Firn” glacier soil and ice metagenomes in Northeast Greenland.

MAG	GenBank assembly accession ID	# contigs	Genome size (bp)	GC %	Compl %	Cont %	Key functional genes												
							puf	puh	bch	acsF	bchE	hemF	hemN	hemJ	hemG	BphP	BphO	RubisCO	Rho-like
ES-bin-14	GCA_014380785.1	633	5,349,535	60.2	79.18	6.59	+	+	+	+	−	+	−	−	−	−	−	−	−
ES-bin-29	GCA_014379705.1	758	5,043,933	67.5	67.91	1.1	−	+	−	−	−	−	−	−	−	−	−	+	−
ES-bin-51	GCA_014379355.1	293	4,821,585	65.3	83.65	2.39	+	+	+	+	−	+	−	−	−	+	+	−	−
ES-bin-78	GCA_014379205.1	339	4,113,156	60.3	89.05	7.69	−	−	−	−	−	−	−	−	−	−	−	−	−
LF-bin-215	GCA_014378185.1	808	3,680,986	65.7	73.65	4.4	−	−	−	−	−	−	−	−	−	−	−	−	+
LF-bin-339	GCA_014377535.1	254	3,787,951	65.7	90.61	3.30	+	+	+	+	−	+	+	−	+	+	+	−	+

*Statistics data were retrieved from a previous study by Zeng et al. (2020). The presence of selected key functional genes was investigated in this study by tBLASTn search in each MAGs using corresponding *G. groenlandica* genes as queries. The BLAST results that pass the threshold of *E* < e-5, coverage >40%, and identity >40%) were regarded as a positive match. +, presence of the gene or at least one gene in the category of *puf* (reaction center-related proteins), *puh* (reaction center assembly proteins) or *bch* (bacteriochlorophyll biosynthesis gene). *acsF*/*bchE*, Mg-protoporphyrin IX monomethyl ester oxidative cyclase (aerobic/anaerobic); *hemF*/*hemN*, coproporphyrinogen oxidase (aerobic/anaerobic); *hemJ*/*hemG*, protoporphyrinogen oxidase (aerobic/anaerobic); *BphP* and *BphO*, bacteriophytochrome; RubisCO, ribulose-1,5-bisphosphate carboxylase/oxygenase; Rho-like, rhodopsin-like. Compl, completeness; Cont, contamination.*

On the AcsF phylogenetic tree (Figure 2B), the two aerobic CGB MAGs, ES-bin-14, and ES-bin-51, were placed on the branches that are distinct from *G. groenlandica* and *G. phototrophica*. However, on the phylogenomic tree (Figure 3), MAGs ES-bin-14, ES-bin-51, and LF-bin-339 are closely related to the members of the *Gemmatimonas* cluster, likely representing novel CGB species in the same genus or family. Four MAGs (GenBank assemblies GCA_007692605.1, GCA_007692505.1, GCA_007692665.1, and GCA_007695195.1) from a high-altitude alkaline salt lake in the Cariboo Plateau in Canada (Zorz et al., 2019) were also found to contain the *pufM* or *acsF* gene and therefore they belong to CGB, albeit distantly related to the *Gemmatimonas* cluster (Figure 3). Intriguingly, MAGs LF-bin-215 and LF-bin-339 also contain a rhodopsin (Rho)-like gene. A proteorhodopsin-like gene is also present in the Gemmatimonadetes MAG Baikal-G1 (non-CGB) assembled from a Lake Baikal metagenome (Cabello-Yeves et al., 2018), potentially providing an additional energy source. However, their function as proton-pump rhodopsins has not yet been verified. Given the low relative abundance of Gemmatimonadetes in the environment, of which only a minor fraction are phototrophic, the ecological function of phototrophic Gemmatimonadetes is likely minor and it is more probable that they only serve as part of a rare microbial biosphere, providing ecosystems with persistent microbial seeds, functional diversity, and ecological resilience (Lynch and Neufeld, 2015; Jousset et al., 2017).

**FIGURE 8:**
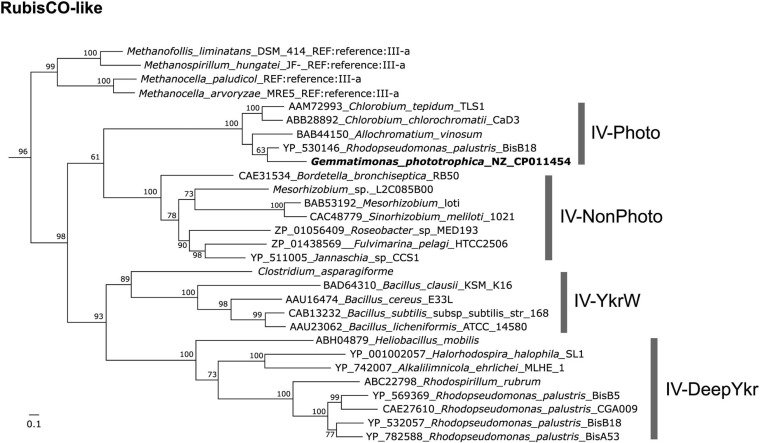
Maximum-likelihood tree of RubisCO-like protein (RLP) gene (GenBank accession no. WP_026848175) in *Gemmatimonas phototrophica*. RubisCO sequences collected and classified by Jaffe et al. (2019) were used as references. Bar represents amino acid residue substitution rate. Bootstrap values between 0.5 and 1 are shown on the branches.

Interestingly, a Gemmatimonadetes MAG (named CSSed162cmB_429) recently assembled from a hypersaline soda lake sediment metagenome (top layer, 0–2 cm) was found to contain genes coding for type-2 RC, type I RubisCO (*rbcLM*), and phosphoribulokinase (*prkB*) (Vavourakis et al., 2019), indicating a photoautotrophic potential in this CGB member. *G. phototrophica* also contains a RubisCO-like gene (GenBank accession no. WP_026848175) but lacks the *prkB* gene, and no RubisCO homolog was found in *G. groenlandica*. Phylogenetic analysis showed that the RubisCO-like gene of *G. phototrophica* groups into the IV-Photo cluster (Tabita et al., 2007) with all members coming from phototrophic Proteobacteria or Chlorobi (Figure 8). It is unclear whether the last common ancestor of the phylum Gemmatimonadetes was a photoautotroph or the photoautotrophic capacity evolved later by HGT. However, this opens a possibility that the evolution of the phylum Gemmatimonadetes might resemble that of the phylum Proteobacteria, where all members were supposed to have originated from a photoautotrophic purple bacterium and the photosynthetic capacity has been lost many times, resulting in various non-photosynthetic lineages (Woese, 1987; Battistuzzi et al., 2004). Over the evolutionary course, *G. groenlandica* may represent aerobic CGB that have adapted to modern fully oxygenated surface environments, whereas *G. phototrophica* represents a more primitive species undergoing evolutionary transitioning from anoxic to oxic environments. More complete genomes from various lineages of CGB are needed to decipher the evolutionary puzzle of phototrophy in this phylum.

## Concluding Remarks

Since our accidental discovery of *G. phototrophica*, the first chlorophototrophic member of the phylum Gemmatimonadetes (Zeng et al., 2014), molecular data accumulated to date suggest CGB are widespread in the environment (Zeng et al., 2016; Cabello-Yeves et al., 2018; Vavourakis et al., 2019). We circumvented the slow growth nature of CGB by employing a target screening strategy that led to the successful isolation of the second member of CGB as a pure culture. The use of antibiotics during the initial enrichment and the selection of a cold low-biomass environment in Greenland for the cultivation attempt appear to be the key to our success. Our strategy also demonstrates the power of combining MALDI-TOF MS and colony infrared imaging techniques in discovering novel chlorophototrophs from nature. The new CGB member of *G. groenlandica* sp. nov. provides an additional model microorganism as a strictly aerobic anoxygenic phototroph in this phylum that readily grows in liquid medium. This trait is not seen in the microaerophilic slow grower *G. phototrophica*, and may prove rather important for future genetic engineering and detailed photophysiological studies.

## Taxonomy

*Gemmatimonas groenlandica* sp. nov. [groen.lan’di.ca. Gr. n. pertaining to the isolation source of Greenland (Groenland in Danish)] is a bacteriochlorophyll *a*-containing bacterium isolated from the stream water in Northeast Greenland. Cells are short to long rods, contain capsule-like structures, and divide in a binary fission mode with budding occasionally observed (Figure 1E). The colonies display a pink-to-red color and cultures turn reddish in stationary phase under fully aerobic conditions. The temperature range for growth is between 15 and 32°C, with optimum at 20–25°C and growth occurs at pH between 6.5 and 9.0 with an optimum at pH 7.3. Cells appear intolerant to NaCl as growth was inhibited even in the presence of 0.1% NaCl. It prefers growth in aerobic conditions but can also grow slowly under microaerophilic conditions (10% O_2_). Fermentative growth was not observed under anaerobic conditions. Growth did not occur under photoautotrophic and chemoautotrophic conditions using sulfide and thiosulfate as electron donor and NaHCO_3_ as the sole carbon source. Chemoorganoheterotrophic and photoheterotrophic growth modes are preferred and various carbon sources are utilized under aerobic, light or dark conditions. Cells are resistant to bacitracin, chloramphenicol, and nystatin but susceptible to neomycin, amoxicillin, tetracycline, and amphotericin B. The substrates utilized as carbon source/electron donor under photo- or chemoheterotrophic condition include saccharin, salicin, adonitol, trehalose, dulcitol, rhamnose, pyruvate, glucose and yeast extract, but not xylose, ribose, erythritol, turanose, cellobiose, melibiose, lyxose, and arabinose. Only yeast extract (0.5 g L^–1^) can be utilized as nitrogen source, but not nitrite, nitrate, glutamine, ammonium ion, and casamino acids. Addition of vitamins is not necessary for growth. The dominant fatty acids are C15:0 iso and C15:1 ω6c (Supplementary Figure 2) and the dominant polar lipids are phosphatidylethanolamine, aminolipid and diphosphatidylglycerol (Supplementary Figure 3). The major respiratory quinones are MK-8 and MK-9 (Supplementary Figure 4). The genomic GC content is 65.1% and the genome size is 5,179,092 bp. The type strain, TET16^T^ (= DSM 110279^T^ and CGMCC 1.18661^T^), was isolated from the surface water of a stream in the Zackenberg Valley in Northeast Greenland.

## Materials and Methods

### Sampling and Screening of Isolates by MALDI-TOF MS

Sampling was done in late August 2017 at a small branch of a stream (74°28′12.5″N, 20°31′02.0″W) close to the Zackenberg Research Station (74°28′10.4″N, 20°34′30.1″W) in Northeast Greenland. This area has a mean annual air temperature of −9.2°C and an annual precipitation of 203 mm with August being the warmest month (mean 5.1°C) (Hasholt and Hagedorn, 2000). The stream is part of the tributaries of the Zackenberg River with very low concentrations of dissolved organic matter (0.6 mg C L^–1^) and soluble reactive phosphate (8.4 μg P L^–1^) (Pastor et al., 2019). Surface water was sampled and filled into a 50 mL bottle which was kept under 4°C until transport to the laboratory in Denmark about 1 month later. The water sample was diluted 1:10 and then 100 μL of dilution was plated onto a 1/5 strength R2A agar plate (Difco). To increase the diversity of cultured bacteria, the following antibiotics were used individually: 8 μg/mL tetracycline (TET), 20 mg L^–1^ piperacillin sodium salt, 20 mL L^–1^ streptomycin, 8 mg L^–1^ gentamicin, and 20 mg L^–1^ kanamycin. For agar plates supplemented with antibiotics, 100 μL of the original water sample was plated. Agar plates were incubated under room temperature and normal laboratory indoor light condition for two to 10 weeks until colonies formed.

To screen for potential phototrophic Gemmatimonadetes bacteria, a two-step strategy was adopted to increase the chance of success with manageable labor efforts: (1), only small (slow growers), pinkish or reddish colonies were considered for MALDI-TOF MS screening based on previous knowledge accumulated from *Gemmatimonas phototrophica* strain AP64, which was isolated from a desert lake (Zeng et al., 2015); (2), the MALDI-TOF MS fingerprints of *G. phototrophica* and *G. aurantiaca* were used as references and only colonies that formed a tight cluster with these two references in the MALDI-TOF MS fingerprinting analysis were considered for further verification as phototrophic Gemmatimonadetes by genome sequencing and measurement of absorption spectra.

All target colonies were subjected to MALDI-TOF MS fingerprinting analysis using the Microflex LT system (Bruker Daltonics, Bremen, Germany) following the procedure described previously (Zervas et al., 2019). Briefly, a toothpick was used to transfer a small amount of a test colony onto the target plate (MSP 96 polished steel, Bruker), which was evenly spread out and formed a thin layer of biomass on the steel plate. The sample was then overlaid with 70% formic acid and allowed for air dry before the addition of 1 μL MALDI-MS matrix solution (α-cyano-4-hydroxycinnamic acid, Sigma-Aldrich). The standard method “MBT_AutoX” was applied to obtain proteome profiles within the mass range of 2 – 20 kDa using the flexControl software (Bruker). The flexAnalysis software (Bruker) was used to smooth the data plot, subtract the baseline and generate main spectra (MSP), followed by a hierarchical clustering analysis using the MALDI Biotyper Compass Explorer software, which produced a dendrogram as output for visual inspection of similarities between samples. For defining different groups at strain/species level, an empirical distance cutoff of 50 was used. There was no consensus on the cutoff at genus or above levels, which varies greatly among different bacterial groups. We used lab-maintained cultures of Proteobacteria as negative controls.

### Genome Sequencing, Phylogeny, and Comparative Genomics

Genomic DNA of the selected isolate TET16^T^ (from a TET-supplemented agar plate) was extracted from cells harvested from 1/5 R2A agar plates after 2–3 weeks growth using the EasyPure bacterial genomic DNA kit (TransGen Biotech, Beijing, China) and was sequenced both on an Illumina NextSeq 500 platform in house and on a PacBio Sequel platform at BGI Hong Kong using a 20K library method for SMART cell with the manufacture’s standard protocols. A total of 3,097,562 Illumina reads (PE 150) and 366,471 PacBio reads were generated. For quality control, the Illumina reads were trimmed at the left end for 10 bases due to irregularities in GC content and at the right end for 30 bases to remove adaptors and irregular bases, while all PacBio reads were used for following *de novo* hybrid-assembly. The gap-free complete genome was assembled using Unicycler (ver. 0.4.8) in a hybrid mode with default settings (Wick et al., 2017). The genome of strain TET16^T^ was annotated with the NCBI’s prokaryotic genome annotation pipeline (GenBank accession no. CP053085).

The 16S rRNA gene and *acsF* gene were retrieved from the TET16 genome and aligned with reference sequences. The 16S rRNA gene reference sequences were downloaded from the NCBI *nr* database through BLASTn search (>97% identities, >1,375 bp, equivalent to >90% coverage). The reference sequences for *acsF* include the sequences downloaded from the NCBI *nr* database through tBLASTn search (>250 amino acids, equivalent to >70% coverage) and those used in two previous studies (Zeng et al., 2016, 2020). For phylogeny inference, sequences were first aligned with MAFFT v7.471 (Katoh and Standley, 2013) using the Q-INS-i algorithm for 16S rRNA genes, which takes secondary structure information of RNA into account and the G-INS-I algorithm for AcsF protein sequences. The most appropriate evolutionary model was determined using ModelTest-NG (Darriba et al., 2020). Then, the phylogenetic tree was built with RAxML-NG (Kozlov et al., 2019) using the nucleotide model GTR + I + G4 for 16S rRNA genes and the amino acid model LG + I + G4 for AcsF protein sequences both with 1,000 bootstrap replicates. The tree was visualized in the Geneious Prime environment (Biomatters, New Zealand).

To demonstrate the ancient connection of phototrophic Gemmatimonadetes bacteria to purple phototrophic bacteria, phylogenetic analysis of the RubisCO-like gene identified in the complete genome of *Gemmatimonas phototrophica* AP64^T^ (GenBank accession no. CP011454; Zeng et al., 2016) but not described before (Zeng et al., 2014) were also performed in this study. The well-classified RubisCO reference sequences were retrieved from the study by Jaffe et al. (2019). Multiple sequence alignment and tree inference were conducted using the same method as described above for the AcsF phylogeny.

The average nucleotide identity (ANI) and average amino acid identity (AAI) between *Gemmatimonas groenlandica* TET16^T^ (this study) and other three type strains in the phylum Gemmatimonadetes that have genome sequences publicly available, including *G. phototrophica* (Zeng et al., 2015), *G. aurantiaca* (Zhang et al., 2003) and *Longimicrobium terrae* (Pascual et al., 2016), were calculated using FastANI (ver. 1.3^2^; Jain et al., 2018) and CompareM (ver. 0.1.1^3^), respectively. The two plasmids in the genome of *Longimicrobium terrae* strain CB-286315 were removed prior to the calculation. Whole genome-level synteny of these four genomes were also calculated using the Easyfig program (ver. 2.2.3; Sullivan et al., 2011) and a circos plot was created with Circa^4^. The synteny of PGCs (∼42 kb) and flanking regions (∼ 30 kb) in *G. groenlandica* and *G. phototrophica* was calculated and visualized using Easyfig.

The unique gene organization features in the PGC of chlorophototrophic Gemmatimonadetes bacteria was evaluated by comparing the two complete PGCs of *G. groenlandica* and *G. phototrophica* and an incomplete but continuous PGC from a Gemmatimonadetes MAG reconstructed from the metagenome of Lake Baikal, Russia (Cabello-Yeves et al., 2018) with various proteobacterial PGCs. The reference PGCs from Proteobacteria were chosen based on tBlastn hits using fused AcsF-BchO-PuhE protein sequences against NCBI’s RefSeq genome database. The reasons for using a fused protein sequence as the query are (1), to select for complete and continuous PGCs, instead of fragmented PGC with parts distantly located on a chromosome as often occurred in purple bacterial genomes (Nagashima and Nagashima, 2013); (2), these three genes’ locations within PGC are more flexible (Zeng and Koblížek, 2017) and thus more susceptible to evolutionary pressure compared to other PGC genes that form sub-clusters and, therefore, they are more likely to reflect species evolution, as has been demonstrated on the *acsF* gene (Boldareva-Nuianzina et al., 2013; Zeng et al., 2014). The top scoring genome from each group at the class level was downloaded and compared to Gemmatimonadetes PGCs.

For the metabolic reconstruction of *G. groenlandica* and *G. phototrophica*, the genomes annotated by NCBI’s Prokaryotic Genome Annotation Pipeline were uploaded to the RAST web server (Aziz et al., 2008) for re-annotation with the original gene prediction information retained. The “Function based Comparison” and “KEGG Metabolic Analysis” functions of RAST were used to analyze both shared and different key metabolic pathways related to a photoheterotrophic life strategy, including central carbon metabolism, energy production, key transporters and membrane structures, and oxidative stress response. The predicted unique functions in one genome were confirmed by tBLASTn searching for homologs in the other genome. If no homologs above the threshold (*E* < e-05, alignment coverage >40%) were found, the gene queried was designated as a unique gene.

The phylogenomic tree of strain TET16^T^ was constructed as follows. The protein FASTA files (^∗^_protein.faa.gz) of all Gemmatimonadetes-affiliated genomes including MAGs and isolates (as of October 2020) were downloaded from the NCBI microbial genomes portal via FTP. The PhyloPhlAn pipeline v3.0.58 (Asnicar et al., 2020) was used to automatically retrieve 400 most universal marker genes from each input genome, multi-align each marker gene, concatenate alignments, and infer the phylogenomic tree. The configuration file for the pipeline was generated using the following command “*phylophlan_write_config_file -d a -o gemma_config.cfg –db_aa diamond –map_dna diamond –map_aa diamond –msa mafft –trim trimal –tree1 fasttree –tree2 raxml –verbose.*” The resulting concatenated alignment includes 36,076 amino acid positions. The genome of *Fibrobacter succinogenes* strain S85 (GenBank assembly no. GCA 000146505.1) was used as the outgroup. The programs FastTree and RAxML (Stamatakis, 2014) were used to build the trees using the PhyloPhlAn 3.0 database in an accurate mode with the diversity level set as medium. Due to high computational cost, bootstrapping on the output RAxML tree was not performed. Instead, the refined phylogeny (the RAxML best tree) produced by RAxML starting from the FastTree phylogeny was selected as the final phylogenomic tree. The tree was edited online at the website of iTOL (Letunic and Bork, 2019). The genome-based taxonomy of TET16^T^ was computed using the command *classify_wf* of the GTDB-Tk tool kit (ver 1.4.0, release R95; Parks et al., 2018).

### Morphology, Phenotypic and Chemotaxonomic Characterization

Strain TET16^T^ grows well on standard R2A agar and in corresponding R2B liquid media. The medium established for optimal growth has been designated as T21 and contains (L^–1^ 0.5 g yeast extract, 0.5 g peptone, 1.0 g K_2_HPO_4_, and 0.5 g pyruvate with a modified SL-8 trace element solution (refer to DSMZ medium 1222) as followed (final conc. L^–1^): 5.2 mg Na_2_-EDTA, 2.09 mg FeSO_4_ × 7 H_2_O, 190 μg CoCl_2_ × 6 H_2_O, 122 μg MnCl_2_ × 4 H_2_O, 70 μg ZnCl_2_, 24 μg NiCl_2_ × 6 H_2_O, 36 μg Na_2_MoO_4_ × 2 H_2_O, 62 μg H_3_BO_3_, 17 μg CuCl_2_ × 2 H_2_O, 266 μg SrCl_2_, 14.7 mg CaCl_2_ × 2 H_2_O, and 20.3 mg MgCl_2_. The pH was adjusted to 7.25–7.3 by addition of 1M HCl solution. The colonies on solid agar plates were observed after 3–5 days of incubation at 23°C aerobically under 12/12 h light/dark regime. Cell imaging was performed using a JEOL JSM-7401F scanning electron microscope (SEM) and a JEOL JEM-1010 transmission electron microscope (TEM) with standard protocols at the Laboratory of Electron Microscopy, Biology Centre of ASCR, České Budějovice, Czechia^5^.

Cell growth of the strain TET16^T^ at different temperatures (4, 10, 15, 18, 20, 22, 25, 30, and 35°C) and pH (4, 5, 6, 7, 8, 9, and 10) was examined using T21 media. The following pH buffer solutions were used: acetic acid/sodium acetate for pH 4-6, K_2_HPO_4_/KH_2_PO_4_ for pH 6-8, sodium bicarbonate/sodium carbonate for pH 9-10. Growth on various NaCl concentrations (0.1, 0.5, 1, 2, 3, 4, and 5) (w/v) was investigated. All the physiological experiments were performed in T21 broth media under 12/12 h light/dark regime. Fermentative growth and anaerobic phototrophic growth was assayed as described previously (Zeng et al., 2015) using T21 media. The microaerobic growth condition (1% or 10% O_2_) was created by purging an anaerobic jar with commercially purchased gases made by mixing air with pure nitrogen gas in a corresponding ratio.

Antibiotics tests were performed on T21 agar plates supplemented with the following antibiotics (25 mg L^–1^ as working concentration unless stated otherise): bacitracin, chloramphenicol, nystatin (100 mg L^–1^), neomycin, amoxicillin, tetracycline (15 mg L^–1^), and amphotericin B (15 mg L^–1^). Carbon source utilization was carried out at 23°C using the carbon-free minimal media containing K_2_HPO_4_ (0.5 g L^–1^), 1 mL trace element solution SL-8 supplemented with one of the following carbon sources (5 mM): saccharin, salicin, adonitol, trehalose, dulcitol, rhamnose, pyruvate, D-glucose, xylose, ribose, erythritol, turanose, cellobiose, melibiose, lyxose, and arabinose. For nitrogen source tests, the minimal media was modified as (L^–1^): 0.5 g K_2_HPO_4_, 0.5 g pyruvate, 1 mL trace element solution SL-8 added with one of following nitrogen sources: NH_4_Cl (5 mM), KNO_3_ (5 mM), glutamine (5 mM), KNO_3_ (5 mM), NaNO_2_ (5 mM), yeast extract (0.5 g L^–1^), and casamino acids (0.5%). For vitamin tests, the T21 medium was supplemented with one of the following vitamins (final conc. L^–1^): biotin (15 μg), folic acid (100 μg), pyridoxin HCl (15 μg), PABA (300 μg), niacin (100 μg), thiamine HCl (500 μg), riboflavin (100 μg), nicotinamide (500 μg), and B12 (15 μg).

BChl *a* fluorescence from colonies in the near infrared region was initially detected with a lab-assembled infra-red colony imaging system as described before (Zeng et al., 2014). The pigment composition was further analyzed and confirmed using high-performance liquid chromatography (HPLC). The cells were harvested from 5 to 6 days old T21 liquid media by centrifugation (10,000 × *g* for 3 min). The pellet was extracted with 100% methanol. 20 μL of the mix was injected into Nexera LC-40 HPLC system (Shimadzu, Japan) equipped with Kinetex 2.6 μm C8 100Å column (150 mm × 4.6 mm, Phenomenex) heated at 40°C. A binary solvent system was used: A, 25% 28 mM ammonium acetate + 75% methanol; B, 100% methanol at a constant flow rate 0.8 mL min^–1^. BChl *a* and carotenoids were observed at 770 and 490 nm, respectively.

Respiratory quinones were extracted with 1 mL 7:2 (vol:vol) aceton:methanol mixture. The debris was removed by 3-min centrifugation in an Eppendorf desktop centrifuge at the top speed. The quinones were analyzed using Prominence-i LC-2030C HPLC system equipped with UV-VIS diode-array detector (Shimadzu Inc., Japan). Respiratory quinones were separated on a heated Luna 3 μm C18(2) 100Å 150 × 4.6 mm column (Phenomenex Inc., United States) using binary solvent system: A – 100% methanol; B – 10:3 methanol/heptane (vol:vol). The eluted quinones were detected at 275 nm and identified based on the retention time and absorption spectra. Natural menaquinones extracted from *Micrococcus luteus*, and purchased ubiquinone-10 were used as control standards. Analysis of polar lipids and fatty acids were carried out by the identification service and Dr. Brian Tindall, at DSMZ (Braunschweig, Germany). The *in vivo* absorption spectra were recorded on a Shimadzu UV2600 spectrophotometer.

### Gemmatimonadetes MAGs From High Arctic Greenland

We previously reported six MAGs of Gemmatimonadetes origin in a metagenomics study of high arctic soil and glacier in Northeast Greenland (Zeng et al., 2020). Detailed analysis of these MAGs other than general description was not carried out in that study. Here we further present the functional genes and phylogenetic data of these Gemmatimonadetes MAGs with the aim to assess potential importance and metabolic diversity of phototrophic Gemmatimonadetes bacteria in Greenlandic environments. The six MAGs include ES-bin-14, ES-bin-29, ES-bin-51, and ES-bin-78 that were assembled from the exposed surface soil metagenome (designated ES) at the “Lille Firn” glacier (designated LF, GPS: 81.566° N, 16.363° W) close to the Villum Research Station in Northeast Greenland and the two bins LF-bin-215 and LF-bin-339 that were assembled from the LF surface ice metagenome (see more details on sampling in Zeng et al., 2020). The genome annotations of the MAGs were downloaded from the NCBI microbial genome portal via FTP.

## Data Availability Statement

The datasets presented in this study can be found in online repositories. The names of the repository/repositories and accession number(s) can be found in the article/Supplementary Material.

## Author Contributions

YZ conceived the study. YZ wrote the manuscript with input from Nupur and MK. MK analyzed the respiratory quinones. Nupur performed the phenotypic and physiological characterization and HPLC with help from YZ and MK. NW carried out the fieldwork. YZ and AM performed the MALDI-TOF MS-related work. Nupur and ATG optimized the cultivation media. YZ and XC assembled and analyzed the genomes and reconstructed the phylogenies. All authors read and approved the final version.

## Conflict of Interest

XC is currently employed at BGI Europe A/S, Denmark. The remaining authors declare that the research was conducted in the absence of any commercial or financial relationships that could be construed as a potential conflict of interest.

## Footnotes

1 https://lpsn.dsmz.de/

2 https://github.com/ParBLiSS/FastANI

3 https://github.com/dparks1134/CompareM

4 http://omgenomics.com/circa

5 http://triton.paru.cas.cz/old-lem

## Supplementary Material

Supplementary Figure 1 Mauve genome alignment of *G. groenlandica* and *G. phototrophica* demonstrating the highly conserved genomic regions housing the photosynthesis gene cluster.

Supplementary Figure 2 DSMZ report on the fatty acids composition in *G. groenlandica* TET16^T^.

Supplementary Figure 3 Polar lipids in *G. groenlandica* TET16^T^ by thin-layer chromatography.

Supplementary Figure 4 Respiratory quinones in *G. groenlandica* TET16^T^ by liquid chromatography.

Supplementary Table 1 Comparison of the genome annotations of *G. groenlandica* and *G. phototrophica*.

## References

[B1] Asnicar F. Thomas A. M. Beghini F. Mengoni C. Manara S. Manghi P. (2020). Precise phylogenetic analysis of microbial isolates and genomes from metagenomes using PhyloPhlAn 3.0. *Nat. Commun.* 11:2500. 10.1038/s41467-020-16366-7 PMC7237447 32427907

[B2] Aziz R. K. Bartels D. Best A. A. DeJongh M. Disz T. Edwards R. A. (2008). The RAST server: rapid annotations using subsystems technology. *BMC Genom.* 9:75. 10.1186/1471-2164-9-75 18261238 PMC2265698

[B3] Battistuzzi F. U. Feijao A. Hedges S. B. (2004). A genomic timescale of prokaryote evolution: insights into the origin of methanogenesis, phototrophy, and the colonization of land. *BMC Evol. Biol.* 4:44. 10.1186/1471-2148-4-44 15535883 PMC533871

[B4] Boldareva-Nuianzina E. N. Bláhová Z. Sobotka R. Koblížek M. (2013). Distribution and origin of oxygen-dependent and oxygen-independent forms of Mg-protoporphyrin monomethylester cyclase among phototrophic *proteobacteria*. *Appl. Environ. Microbiol.* 79 2596–2604. 10.1128/aem.00104-13 23396335 PMC3623192

[B5] Cabello-Yeves P. J. Zemskaya T. I. Rosselli R. Coutinho F. H. Zakharenko A. S. Blinov V. V. (2018). Genomes of novel microbial lineages assembled from the sub-ice waters of Lake Baikal. *Appl. Environ. Microbiol.* 84:e02132-17. 10.1128/AEM.02132-17 PMC5734018 29079621

[B6] Chee-Sanford J. Tian D. Sanford R. (2019). Consumption of N2O and other N-cycle intermediates by *Gemmatimonas aurantiaca* strain T-27. *Microbiol. SGM* 165 1345–1354. 10.1099/mic.0.000847 31580255

[B7] Chun J. Oren A. Ventosa A. Christensen H. Arahal D. R. da Costa M. S. (2018). Proposed minimal standards for the use of genome data for the taxonomy of prokaryotes. *Int. J. Syst. Evol. Microbiol.* 68 461–466. 10.1099/ijsem.0.002516 29292687

[B8] Dachev M. Bína D. Sobotka R. Moravcová L. Gardian Z. Kaftan D. (2017). Unique double concentric ring organization of light harvesting complexes in *Gemmatimonas phototrophica*. *PLoS Biol.* 15:e2003943. 10.1371/journal.pbio.2003943 29253871 PMC5749889

[B9] Darriba D. Posada D. Kozlov A. M. Stamatakis A. Morel B. Flouri T. (2020). ModelTest-NG: a new and scalable tool for the selection of DNA and protein evolutionary models. *Mol. Biol. Evol.* 37 291–294. 10.1093/molbev/msz189 31432070 PMC6984357

[B10] DeBruyn J. M. Nixon L. T. Fawaz M. N. Johnson A. M. Radosevich M. (2011). Global biogeography and quantitative seasonal dynamics of gemmatimonadetes in soil. *Appl. Environ. Microbiol.* 77 6295–6300. 10.1128/aem.05005-11 21764958 PMC3165389

[B11] Hanada S. Sekiguchi Y. (2014). “The phylum gemmatimonadetes,” in *The Prokaryotes – Other Major Lineages of Bacteria and the Archaea*, eds Rosenberg E. DeLong E. F. Lory S. Stackebrandt E. Thompson F. (Berlin: Springer), 677–681. 10.1007/978-3-642-38954-2_164

[B12] Hasholt B. Hagedorn B. (2000). Hydrology and geochemistry of river-borne material in a high arctic drainage system, Zackenberg, Northeast Greenland. *Arctic Antarct. Alpine Res.* 32 84–94. 10.1080/15230430.2000.12003342

[B13] Jaffe A. L. Castelle C. J. Dupont C. L. Banfield J. F. (2019). Lateral gene transfer shapes the distribution of RuBisCO among candidate phyla radiation bacteria and DPANN archaea. *Mol. Biol. Evol.* 36 435–446. 10.1093/molbev/msy234 30544151 PMC6389311

[B14] Jain C. Rodriguez-R L. M. Phillippy A. M. Konstantinidis K. T. Aluru S. (2018). High throughput ANI analysis of 90K prokaryotic genomes reveals clear species boundaries. *Nat. Commun.* 9 1–8. 30504855 10.1038/s41467-018-07641-9 PMC6269478

[B15] Janssen P. H. (2006). Identifying the dominant soil bacterial taxa in libraries of 16S rRNA and 16S rRNA genes. *Appl. Environ. Microbiol.* 72 1719–1728. 10.1128/aem.72.3.1719-1728.2006 16517615 PMC1393246

[B16] Jousset A. Bienhold C. Chatzinotas A. Gallien L. Gobet A. Kurm V. (2017). Where less may be more: how the rare biosphere pulls ecosystems strings. *ISME J.* 11 853–862. 10.1038/ismej.2016.174 28072420 PMC5364357

[B17] Kato K. Tanaka R. Sano S. Tanaka A. Hosaka H. (2010). Identification of a gene essential for protoporphyrinogen IX oxidase activity in the *Cyanobacterium synechocystis* sp. *PCC*6803. *Proc. Natl. Acad. Sci. U.S.A.* 107 16649–16654. 10.1073/pnas.1000771107 20823222 PMC2944763

[B18] Katoh K. Standley D. M. (2013). MAFFT multiple sequence alignment software version 7: improvements in performance and usability. *Mol. Biol. Evol.* 30 772–780. 10.1093/molbev/mst010 23329690 PMC3603318

[B19] Kim M. Oh H. S. Park S. C. Chun J. (2014). Towards a taxonomic coherence between average nucleotide identity and 16S rRNA gene sequence similarity for species demarcation of prokaryotes. *Int. J. Syst. Evol. Microbiol.* 64 346–351. 10.1099/ijs.0.059774-0 24505072

[B20] Koblížek M. (2015). Ecology of aerobic anoxygenic phototrophs in aquatic environments. *FEMS Microbiol. Rev.* 39 854–870. 10.1093/femsre/fuv032 26139241

[B21] Koblížek M. Dachev M. Bína D. Nupur L. Piwosz K. Kaftan D. (2020). Utilization of light energy in phototrophic Gemmatimonadetes. *J. Photochem. Photobiol. B*. 213:112085. 10.1016/j.jphotobiol.2020.112085 33220599

[B22] Kozlov A. M. Darriba D. Flouri T. Morel B. Stamatakis A. (2019). RAxML-NG: a fast, scalable and user-friendly tool for maximum likelihood phylogenetic inference. *Bioinformatics* 35 4453–4455. 10.1093/bioinformatics/btz305 31070718 PMC6821337

[B23] Lagier J. C. Dubourg G. Million M. Cadoret F. Bilen M. Fenollar F. (2018). Culturing the human microbiota and culturomics. *Nat. Rev. Microbiol.* 16 540–550. 10.1038/s41579-018-0041-0 29937540

[B24] Letunic I. Bork P. (2019). Interactive tree of life (iTOL) v4: recent updates and new developments. *Nucleic Acids Res.* 47 W256–W259. 30931475 10.1093/nar/gkz239 PMC6602468

[B25] Lynch M. D. Neufeld J. D. (2015). Ecology and exploration of the rare biosphere. *Nat. Rev. Microbiol.* 13 217–229. 10.1038/nrmicro3400 25730701

[B26] Nagashima S. Nagashima K. V. (2013). Comparison of photosynthesis gene clusters retrieved from total genome sequences of purple bacteria. *Adv. Bot. Res.* 66 151–178. 10.1016/b978-0-12-397923-0.00005-9

[B27] Park D. Kim H. Yoon S. (2017). Nitrous oxide reduction by an obligate aerobic bacterium, *Gemmatimonas aurantiaca* strain T-27. *Appl. Environ. Microbiol.* 83 e502–e517. 10.1128/AEM.00502-17 PMC5452805 28389533

[B28] Parks D. H. Chuvochina M. Waite D. W. Rinke C. Skarshewski A. Chaumeil P. A. (2018). A standardized bacterial taxonomy based on genome phylogeny substantially revises the tree of life. *Nat. Biotechnol.* 36 996–1004. 10.1038/nbt.4229 30148503

[B29] Pascual J. Foesel B. U. Geppert A. Huber K. J. Boedeker C. Luckner M. (2018). *Roseisolibacter agri* gen. nov., sp nov., a novel slow-growing member of the under-represented phylum Gemmatimonadetes. *Intern. J. Syst. Evol. Microbiol.* 68 1028–1036. 10.1099/ijsem.0.002619 29458671

[B30] Pascual J. Garcia-Lopez M. Bills G. F. Genilloud O. (2016). *Longimicrobium terrae* gen. nov., sp nov., an *Oligotrophic bacterium* of the under-represented phylum Gemmatimonadetes isolated through a system of miniaturized diffusion chambers. *Intern. J. Syst. Evol. Microbiol.* 66 1976–1985. 10.1099/ijsem.0.000974 26873585

[B31] Pastor A. Freixa A. Skovsholt L. J. Wu N. Romaní A. M. Riis T. (2019). Microbial organic matter utilization in high-arctic streams: key enzymatic controls. *Microb. Ecol.* 78 539–554. 10.1007/s00248-019-01330-w 30739147

[B32] Rosselló-Móra R. Amann R. (2015). Past and future species definitions for Bacteria and Archaea. *Syst. Appl. Microbiol.* 38 209–216. 10.1016/j.syapm.2015.02.001 25747618

[B33] Stamatakis A. (2014). RAxML version 8: a tool for phylogenetic analysis and post-analysis of large phylogenies. *Bioinformatics* 30 1312–1313. 10.1093/bioinformatics/btu033 24451623 PMC3998144

[B34] Sullivan M. J. Petty N. K. Beatson S. A. (2011). Easyfig: a genome comparison visualizer. *Bioinformatics* 27 1009–1010. 10.1093/bioinformatics/btr039 21278367 PMC3065679

[B35] Tabita F. R. Hanson T. E. Li H. Satagopan S. Singh J. Chan S. (2007). Function, structure, and evolution of the RubisCO-like proteins and their RubisCO homologs. *Microbiol. Mol. Biol. Rev.* 71 576–599. 10.1128/mmbr.00015-07 18063718 PMC2168653

[B36] Tahon G. Willems A. (2017). Isolation and characterization of aerobic anoxygenic phototrophs from exposed soils from the Sof. Rondane Mountains, East Antarctica. *Syst. Appl. Microbiol.* 40 357–369. 10.1016/j.syapm.2017.05.007 28705596

[B37] Vavourakis C. D. Mehrshad M. Balkema C. Van Hall R. Andrei A. Ş. Ghai R. (2019). Metagenomes and metatranscriptomes shed new light on the microbial-mediated sulfur cycle in a Siberian soda lake. *BMC Biol.* 17:69. 10.1186/s12915-019-0688-7 31438955 PMC6704655

[B38] Wick R. R. Judd L. M. Gorrie C. L. Holt K. E. (2017). Unicycler: resolving bacterial genome assemblies from short and long sequencing reads. *PLoS Comput. Biol.* 13:e1005595. 10.1371/journal.pcbi.1005595 28594827 PMC5481147

[B39] Wilke A. Bischof J. Gerlach W. Glass E. Harrison T. Keegan K. P. (2016). The MG-RAST metagenomics database and portal in 2015. *Nucleic Acids Res.* 44 D590–D594. 10.1007/8623_2015_119 26656948 PMC4702923

[B40] Woese C. R. (1987). Bacterial evolution. *Microbiol. Rev.* 51:221. 10.1128/mr.51.2.221-271.1987 PMC373105 2439888

[B41] Youssef N. H. Elshahed M. S. (2009). Diversity rankings among bacterial lineages in soil. *ISME J.* 3 305–313. 10.1038/ismej.2008.106 18987677

[B42] Zeng Y. Baumbach J. Barbosa E. G. V. Azevedo V. Zhang C. Koblížek M. (2016). Metagenomic evidence for the presence of phototrophic Gemmatimonadetes bacteria in diverse environments. *Environ. Microbiol. Rep.* 8 139–149. 10.1111/1758-2229.12363 26636755

[B43] Zeng Y. Chen H. Madsen A. M. Zervas A. Nielsen T. K. Andrei A. (2020). Potential rhodopsin and bacteriochlorophyll-based dual phototrophy in a high Arctic glacier. *mBio* 11:e02641-20. 10.1128/mBio.02641-20 33234687 PMC7701988

[B44] Zeng Y. Feng F. Medová H. Dean J. Koblížek M. (2014). Functional type 2 photosynthetic reaction centers found in the rare bacterial phylum Gemmatimonadetes. *Proc. Natl. Acad. Sci. U.S.A.* 111 7795–7800. 10.1073/pnas.1400295111 24821787 PMC4040607

[B45] Zeng Y. Koblížek M. (2017). “Phototrophic Gemmatimonadetes: a new “purple” branch on the bacterial tree of life,” in *Modern Topics in the Phototrophic Prokaryotes*, ed. Hallenbeck P. C. (Cham: Springer), 163–192. 10.1007/978-3-319-46261-5_5

[B46] Zeng Y. Selyanin V. Lukeš M. Dean J. Kaftan D. Feng F. (2015). Characterization of the microaerophilic, bacteriochlorophyll a-containing bacterium *Gemmatimonas phototrophica* sp. nov., and emended descriptions of the genus *Gemmatimonas* and *Gemmatimonas aurantiaca*. *Intern. J. Syst. Evol. Microbiol.* 65 2410–2419. 10.1099/ijs.0.000272 25899503

[B47] Zervas A. Zeng Y. Madsen A. M. Hansen L. H. (2019). Genomics of aerobic photoheterotrophs in wheat phyllosphere reveals divergent evolutionary patterns of photosynthetic genes in *Methylobacterium* spp. *Genome Biol. Evol.* 11 2895–2908. 10.1093/gbe/evz204 31626703 PMC6798729

[B48] Zhang H. Sekiguchi Y. Hanada S. Hugenholtz P. Kim H. Kamagata Y. (2003). *Gemmatimonas aurantiaca* gen. nov., sp nov., a gram-negative, aerobic, polyphosphate-accumulating micro-organism, the first cultured representative of the new bacterial phylum *Gemmatimonadetes* phyl. nov. *Intern. J. Syst. Evol. Microbiol.* 53 1155–1163. 10.1099/ijs.0.02520-0 12892144

[B49] Zorz J. K. Sharp C. Kleiner M. Gordon P. M. Pon R.T. Dong X. (2019). A shared core microbiome in soda lakes separated by large distances. *Nat. Commun.* 10 1–10. 31530813 10.1038/s41467-019-12195-5 PMC6748926

