# Supplementary material for: Gemmatimonas groenlandica sp. nov. Is an Aerobic Anoxygenic Phototroph in the Phylum Gemmatimonadetes
Source: Front Microbiol. 2021 Jan 15;11:606612. doi: 10.3389/fmicb.2020.606612 (PMC7844134; doi:10.3389/fmicb.2020.606612)

Figure S1 Mauve genome alignment of *G. groenlandica* and *G. phototrophica* demonstrating the highly conserved genomic regions housing the photosynthesis gene cluster (PGC).

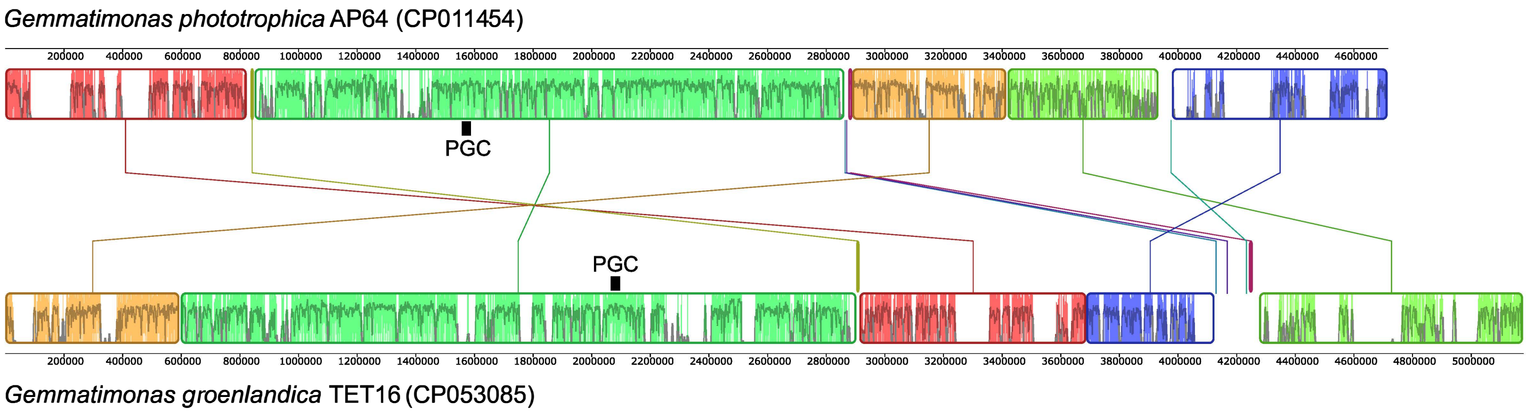

Supplement: Supplementary Figure 1 — Mauve genome alignment of G. groenlandica and G. phototrophica demonstrating the highly conserved genomic regions housing the photosynthesis gene cluster. [file Image_1.PDF]
