# Supplementary material for: Gemmatimonas groenlandica sp. nov. Is an Aerobic Anoxygenic Phototroph in the Phylum Gemmatimonadetes
Source: Front Microbiol. 2021 Jan 15;11:606612. doi: 10.3389/fmicb.2020.606612 (PMC7844134; doi:10.3389/fmicb.2020.606612)
Supplement: Supplementary Figure 2 — DSMZ report on the fatty acids composition in G. groenlandica TET16T. [file Image_2.PDF]

Figure S2 DSMZ report on the fatty acid composition in *G. groenlandica*.

Volume: DATA File: E197045.49A Samp Ctr: 11 ID Number: 7348
Type: Samp Bottle: 18 Method: TSBA40
Created: 7/4/2019 5:18:23 PM
Sample ID: UN-V-19-385-TET16-NUPUR(15mg ZM)

RT	Response	Ar/Ht	RFact	ECL	Peak Name	Percent	Comment1	Comment2
1.631	3.694E+8	0.026	----	7.018	SOLVENT PEAK	----	< min rt	
3.045	283	0.028	----	9.839		----		
4.373	3477	0.028	1.069	11.608	12:0 ISO	0.99	ECL deviates -0.001	Reference -0.004
4.730	299	0.029	1.052	11.999	12:0	0.08	ECL deviates -0.001	Reference -0.005
5.433	25010	0.031	1.029	12.615	13:0 ISO	6.82	ECL deviates 0.001	Reference -0.004
5.534	723	0.032	1.026	12.703	13:0 ANTEISO	0.20	ECL deviates 0.001	Reference -0.003
5.875	462	0.031	1.016	13.001	13:0	0.12	ECL deviates 0.001	Reference -0.003
6.591	650	0.038	----	13.526		----		
6.717	22859	0.035	0.997	13.619	14:0 ISO	6.04	ECL deviates 0.000	Reference -0.005
6.790	390	0.031	----	13.672		----		
6.923	687	0.036	----	13.770		----		
7.104	394	0.033	0.989	13.902	14:1 w5c	0.10	ECL deviates 0.001	
7.236	4290	0.036	0.987	13.999	14:0	1.12	ECL deviates -0.001	Reference -0.006
7.837	777	0.035	0.977	14.391	ISO 15:1 AT 5	0.20	ECL deviates 0.002	
7.966	4986	0.041	0.975	14.475	Sum In Feature 1	1.29	ECL deviates -0.003	15:1 ISO I/13:0 3OH
8.061	656	0.033	0.974	14.536	15:1 ANTEISO A	0.17	ECL deviates 0.009	
8.196	100986	0.038	0.972	14.624	15:0 ISO	26.02	ECL deviates 0.001	Reference -0.003
8.333	2531	0.039	0.970	14.714	15:0 ANTEISO	0.65	ECL deviates 0.001	Reference -0.004
8.409	15850	0.040	----	14.763		----		
8.553	44151	0.039	0.967	14.857	15:1 w6c	11.32	ECL deviates 0.001	
8.773	14494	0.039	0.964	15.000	15:0	3.70	ECL deviates 0.000	Reference -0.004
8.975	5980	0.041	0.962	15.121	14:0 ISO 3OH	1.52	ECL deviates 0.002	
9.419	11800	0.040	0.957	15.387	16:1 w7c alcohol	2.99	ECL deviates 0.000	
9.538	32636	0.046	0.955	15.459	16:1 ISO H	8.27	ECL deviates -0.002	
9.815	17196	0.043	0.953	15.625	16:0 ISO	4.34	ECL deviates -0.002	Reference -0.005
10.033	4246	0.041	0.951	15.756	16:1 w11c	1.07	ECL deviates -0.001	
10.132	64417	0.042	0.950	15.815	Sum In Feature 3	16.22	ECL deviates -0.007	16:1 w7c/15 iso 2OH
10.282	1439	0.042	0.948	15.905	16:1 w5c	0.36	ECL deviates -0.004	
10.432	3410	0.042	0.947	15.995	16:0	0.86	ECL deviates -0.005	Reference -0.007
10.665	6962	0.041	0.945	16.130	15:0 ISO 3OH	1.74	ECL deviates -0.004	
11.157	5771	0.043	0.942	16.414	ISO 17:1 w9c	1.44	ECL deviates -0.002	
11.303	2251	0.051	0.941	16.498	15:0 3OH	0.56	ECL deviates -0.005	
11.539	1124	0.051	0.939	16.634	17:0 ISO	0.28	ECL deviates 0.004	Reference 0.004
11.806	2724	0.045	0.938	16.788	17:1 w8c	0.68	ECL deviates -0.004	
11.926	1455	0.043	0.937	16.857	17:1 w6c	0.36	ECL deviates -0.003	
12.417	556	0.043	0.934	17.138	16:0 ISO 3OH	0.14	ECL deviates -0.012	
12.900	615	0.045	0.932	17.411	17:0 10 methyl	0.15	ECL deviates 0.002	
13.516	526	0.045	----	17.759		----		
13.613	716	0.042	0.929	17.814	18:1 w7c	0.18	ECL deviates -0.009	
13.751	2286	0.046	----	17.898		----		
----	4986	---	----	----	Summed Feature 1	1.29	15:1 ISO H/13:0 3OH	13:0 3OH/15:1 i I/H
----	----	---	----	----		----	15:1 ISO I/13:0 3OH	
----	64417	---	----	----	Summed Feature 3	16.22	16:1 w7c/15 iso 2OH	15:0 ISO 2OH/16:1w7c

ECL Deviation: 0.004 Reference ECL Shift: 0.004 Number Reference Peaks: 13
Total Response: 410063 Total Named: 389392
Percent Named: 94.96% Total Amount: 377162

*** No Matches found in TSBA40

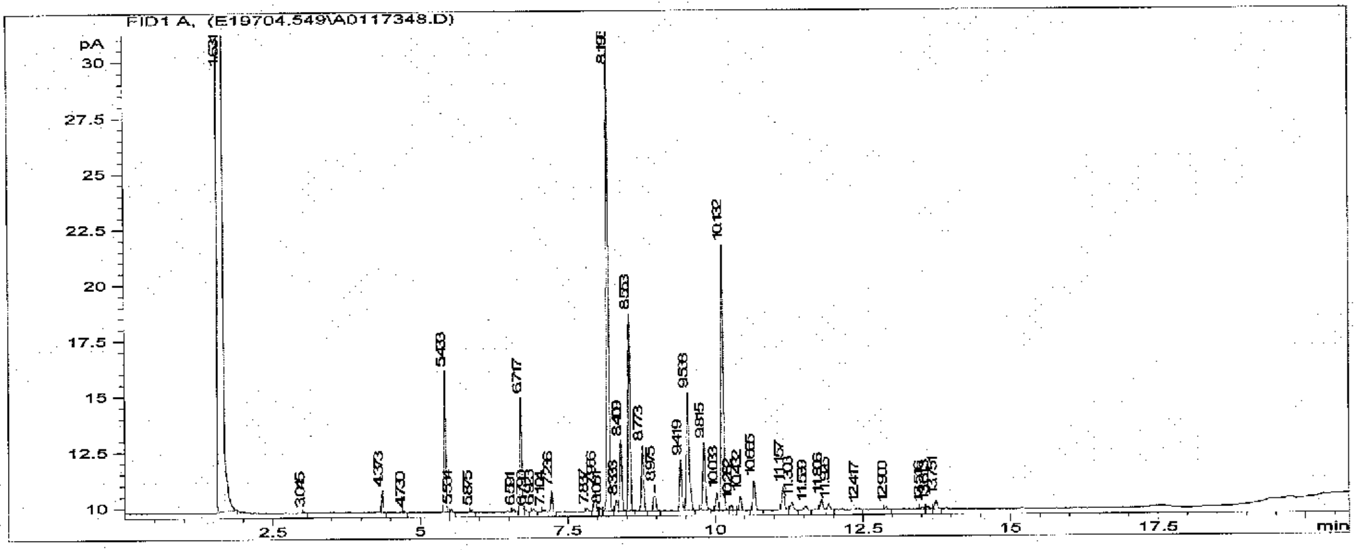
FID1 A, (E19704.549\A0117348.D)
pA
min

Volume: DATA File: E197045.49A Samp Ctr: 11 ID Number: 7348
Type: Samp Bottle: 18 Method: TSBA40 Calc. Method: ANAER6
Created: 7/4/2019 5:18:23 PM
Sample ID: UN-V-19-385-TET16-NUPUR(15mg ZM)

RT	Response	Ar/Ht	RFact	ECL	Peak Name	Percent	Comment1	Comment2
1.631	3.694E+8	0.026	----	7.019	SOLVENT PEAK	----	< min rt	
3.045	283	0.028	----	9.839		----		
4.373	3477	0.028	1.069	11.608	12:0 ISO FAME	1.10	ECL deviates 0.000	Reference -0.003
4.730	299	0.029	1.052	11.999	12:0 FAME	0.09	ECL deviates -0.001	Reference -0.005
5.433	25010	0.031	1.029	12.614	13:0 ISO FAME	7.63	ECL deviates 0.000	Reference -0.004
5.534	723	0.032	1.026	12.703	13:0 ANTEISO FAME	0.22	ECL deviates 0.000	Reference -0.004
5.875	462	0.031	1.016	13.001	13:0 FAME	0.14	ECL deviates 0.001	Reference -0.003
6.591	650	0.038	----	13.526		----		
6.717	22859	0.035	0.997	13.618	14:0 ISO FAME	6.75	ECL deviates 0.000	Reference -0.004
6.790	390	0.031	----	13.671		----		
6.923	687	0.036	----	13.769		----		
7.104	394	0.033	0.989	13.901	14:1 CIS 9 FAME	0.12	ECL deviates -0.001	
7.236	4290	0.036	0.987	13.998	14:0 FAME	1.25	ECL deviates -0.002	Reference -0.006
7.837	777	0.035	----	14.390		----		
7.966	4986	0.041	0.975	14.474	14:0 DMA	1.44	ECL deviates 0.002	Reference -0.002
8.061	656	0.033	----	14.535		----		
8.196	100986	0.038	0.972	14.623	15:0 ISO FAME	29.08	ECL deviates 0.000	Reference -0.003
8.333	2531	0.039	0.970	14.713	15:0 ANTEISO FAME	0.73	ECL deviates -0.001	Reference -0.005
8.409	15850	0.040	0.969	14.762	Sum In Feature 4	4.55	ECL deviates 0.000	UN 14.762 15:2 ? FA
8.553	44151	0.039	0.967	14.855	15:1 CIS 9/t 8 FAME	12.65	ECL deviates -0.002	
8.773	14494	0.039	0.964	14.999	15:0 FAME	4.14	ECL deviates -0.001	Reference -0.004
8.975	5980	0.041	0.962	15.120	15:0 ISO DMA	1.70	ECL deviates 0.008	Reference 0.005
9.419	11800	0.040	----	15.386		----		
9.538	32636	0.046	----	15.458		----		
9.815	17196	0.043	0.953	15.624	16:0 ISO FAME	4.86	ECL deviates -0.003	Reference -0.005
10.033	4246	0.041	----	15.755		----		
10.132	64417	0.042	0.950	15.814	16:1 CIS 9 FAME	18.13	ECL deviates -0.004	
10.282	1439	0.042	0.948	15.904	16:1 CIS 11 FAME	0.40	ECL deviates -0.005	
10.432	3410	0.042	0.947	15.994	16:0 FAME	0.96	ECL deviates -0.006	Reference -0.007
10.665	6962	0.041	0.945	16.129	15:0 ISO 3OH FAME	1.95	ECL deviates -0.006	
11.157	5771	0.043	----	16.413		----		
11.303	2251	0.051	0.941	16.497	15:0 3OH FAME	0.63	ECL deviates -0.009	
11.539	1124	0.051	0.939	16.633	17:0 ISO FAME	0.31	ECL deviates 0.003	Reference 0.004
11.806	2724	0.045	0.937	16.787	Sum In Feature 8	0.76	ECL deviates -0.007	17:1 CIS 9 FAME
11.926	1455	0.043	0.937	16.857	17:1 CIS 11 FAME	0.40	ECL deviates -0.007	
12.417	556	0.043	----	17.138		----		
12.900	615	0.045	----	17.411		----		
13.516	526	0.045	----	17.759		----		
13.613	716	0.042	----	17.814		----		
13.751	2286	0.046	----	17.896		----		
----	15850	---	----	----	Summed Feature 4	4.55	UN 14.762 15:2 ? FA	15:2 FAME
----	-----	---	----	----		----	15:1 CIS 7	
----	2724	---	----	----	Summed Feature 8	0.76	17:1 CIS 9 FAME	17:2 FAME @ 16.801

ECL Deviation: 0.004 Reference ECL Shift: 0.004 Number Reference Peaks: 15
Total Response: 410063 Total Named: 347470
Percent Named: 84.74% Total Amount: 337422

*** No Matches found in BHIBLA

Volume: DATA File: E197045.49A Samp Ctr: 11 ID Number: 7348
Type: Samp Bottle: 18 Method: TSBA40 Calc. Method: TSBA6
Created: 7/4/2019 5:18:23 PM
Sample ID: UN-V-19-385-TET16-NUPUR(15mg ZM)

RT	Response	Ar/Ht	RFact	ECL	Peak Name	Percent	Comment1	Comment2
1.631	3.694E+8	0.026	----	7.018	SOLVENT PEAK	----	< min rt	
3.045	283	0.028	----	9.839		----		
4.373	3477	0.028	1.066	11.608	12:0 iso	1.02	ECL deviates -0.001	Reference -0.004
4.730	299	0.029	1.050	11.999	12:0	0.09	ECL deviates -0.001	Reference -0.005
5.433	25010	0.031	1.027	12.615	13:0 iso	7.08	ECL deviates 0.001	Reference -0.004
5.534	723	0.032	1.024	12.703	13:0 anteiso	0.20	ECL deviates 0.001	Reference -0.003
5.875	462	0.031	1.014	13.001	13:0	0.13	ECL deviates 0.001	Reference -0.003
6.591	650	0.038	----	13.526		----		
6.717	22859	0.035	0.995	13.619	14:0 iso	6.27	ECL deviates 0.000	Reference -0.005
6.790	390	0.031	----	13.672		----		
6.923	687	0.036	----	13.770		----		
7.104	394	0.033	0.987	13.902	14:1 w5c	0.11	ECL deviates 0.001	
7.236	4290	0.036	0.985	13.999	14:0	1.17	ECL deviates -0.001	Reference -0.006
7.837	777	0.035	0.975	14.391	15:1 iso w9c	0.21	ECL deviates 0.002	
7.966	4986	0.041	0.973	14.475	Sum In Feature 1	1.34	ECL deviates -0.003	13:0 3OH/15:1 i H
8.061	656	0.033	0.972	14.536	15:1 anteiso A	0.18	ECL deviates 0.009	
8.196	100986	0.038	0.970	14.624	15:0 iso	27.02	ECL deviates 0.001	Reference -0.003
8.333	2531	0.039	0.968	14.714	15:0 anteiso	0.68	ECL deviates 0.001	Reference -0.004
8.409	15850	0.040	----	14.763		----		
8.553	44151	0.039	0.965	14.857	15:1 w6c	11.75	ECL deviates 0.001	
8.773	14494	0.039	----	15.000	15:0	----	ECL deviates 0.000	
8.975	5980	0.041	0.960	15.121	14:0 iso 3OH	1.58	ECL deviates 0.002	
9.419	11800	0.040	0.955	15.387	16:1 w7c alcohol	3.11	ECL deviates 0.000	
9.538	32636	0.046	0.954	15.459	16:1 iso H	8.59	ECL deviates -0.002	
9.815	17196	0.043	0.951	15.625	16:0 iso	4.51	ECL deviates -0.002	Reference -0.005
10.033	4246	0.041	0.949	15.756	16:1 w11c	1.11	ECL deviates -0.001	
10.132	64417	0.042	0.948	15.815	Sum In Feature 3	16.85	ECL deviates -0.007	16:1 w7c/16:1 w6c
10.282	1439	0.042	0.947	15.905	16:1 w5c	0.38	ECL deviates -0.004	
10.432	3410	0.042	0.945	15.995	16:0	0.89	ECL deviates -0.005	Reference -0.007
10.665	6962	0.041	0.944	16.130	15:0 iso 3OH	1.81	ECL deviates -0.004	
11.157	5771	0.043	0.940	16.414	Sum In Feature 9	1.50	ECL deviates -0.002	17:1 iso w9c
11.303	2251	0.051	0.939	16.498	15:0 3OH	0.58	ECL deviates -0.005	
11.539	1124	0.051	0.938	16.634	17:0 iso	0.29	ECL deviates 0.004	Reference 0.004
11.806	2724	0.045	0.936	16.788	17:1 w8c	0.70	ECL deviates -0.004	
11.926	1455	0.043	0.935	16.857	17:1 w6c	0.38	ECL deviates -0.003	
12.417	556	0.043	0.932	17.138	16:0 iso 3OH	0.14	ECL deviates -0.012	
12.900	615	0.045	0.930	17.411	17:0 10-methyl	0.16	ECL deviates 0.002	
13.516	526	0.045	----	17.759		----		
13.613	716	0.042	0.928	17.814	Sum In Feature 8	0.18	ECL deviates -0.009	18:1 w7c
13.751	2286	0.046	----	17.898		----		
----	4986	---	----	----	Summed Feature 1	1.34	15:1 iso H/13:0 3OH	13:0 3OH/15:1 i H
----	64417	---	----	----	Summed Feature 3	16.85	16:1 w7c/16:1 w6c	16:1 w6c/16:1 w7c
----	716	---	----	----	Summed Feature 8	0.18	18:1 w7c	18:1 w6c
----	5771	---	----	----	Summed Feature 9	1.50	17:1 iso w9c	16:0 10-methyl

ECL Deviation: 0.004 Reference ECL Shift: 0.004 Number Reference Peaks: 12
Total Response: 395569 Total Named: 374898
Percent Named: 94.77% Total Amount: 362551

*** No Matches found in TSBA6
