# Supplementary material for: Gemmatimonas groenlandica sp. nov. Is an Aerobic Anoxygenic Phototroph in the Phylum Gemmatimonadetes
Source: Front Microbiol. 2021 Jan 15;11:606612. doi: 10.3389/fmicb.2020.606612 (PMC7844134; doi:10.3389/fmicb.2020.606612)
Supplement: Supplementary Figure 3 — Polar lipids in G. groenlandica TET16T by thin-layer chromatography. [file Image_3.PDF]

Figure S3 Polar lipids in *G. groenlandica* by thin-layer chromatography.

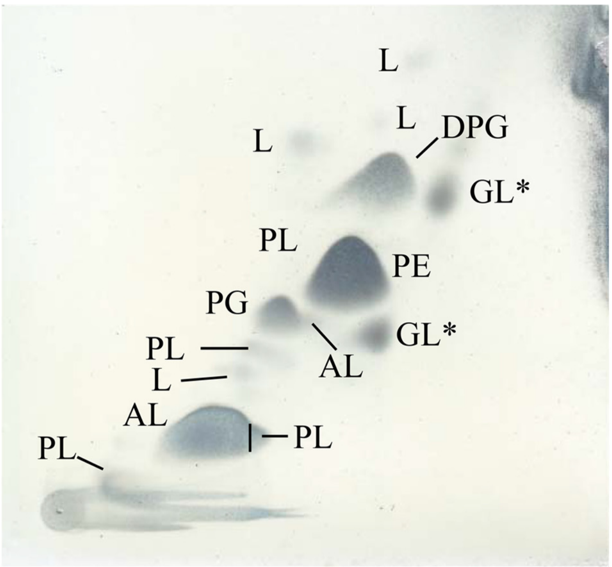

L = Lipid

AL = Aminolipid

PL = Phospholipid

PG = Phosphatidylglycerol

GL* = Glycolipid and pigment

PE = Phosphatidylethanolamine

DPG = Diphosphatidylglycerol
