# Supplementary material for: Gemmatimonas groenlandica sp. nov. Is an Aerobic Anoxygenic Phototroph in the Phylum Gemmatimonadetes
Source: Front Microbiol. 2021 Jan 15;11:606612. doi: 10.3389/fmicb.2020.606612 (PMC7844134; doi:10.3389/fmicb.2020.606612)

Figure S4 Respiratory quinones in *G. groenlandica.* The well-characterized *Micrococcus luteus* was used as reference.

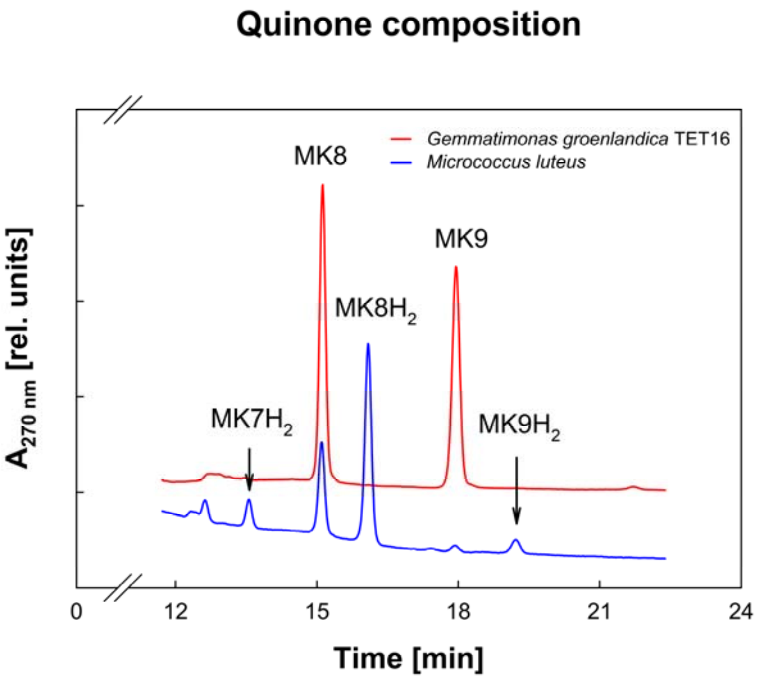

Supplement: Supplementary Figure 4 — Respiratory quinones in G. groenlandica TET16T by liquid chromatography. [file Image_4.PDF]
